# Supplementary material for: Occupational prestige and sickness absence inequality in employed women and men in Sweden: a registry-based study
Source: BMJ Open. 2021 Jun 8;11(6):e050191. doi: 10.1136/bmjopen-2021-050191 (PMC8190050; doi:10.1136/bmjopen-2021-050191)
Supplement: Supplementary data [file bmjopen-2021-050191supp001.pdf]

Online supplementary table A1. Gender-stratified association between occupational prestige and number of sickness absence days at follow-up. Incidence rate ratios (IRR) and 95% confidence intervals (95%CI) obtained from generalized estimating equation (GEE) with negative binomial regression

		Age-adjusted IRR (95%CI)	Model I IRR (95%CI)	Model II IRR (95%CI)	Model III IRR (95%CI)	Model IV IRR (95%CI)	Model V IRR (95%CI)
Women	N = 48029 weighted %						
Occupational prestige							
Low	34.8	2.05 (2.02-2.08)	2.06 (2.03-2.09)	1.85 (1.82-1.88)	1.48 (1.45-1.51)	1.24 (1.19-1.28)	1.22 (1.18-1.27)
Medium	32.1	1.21 (1.19-1.23)	1.19 (1.18-1.21)	1.12 (1.11-1.14)	1.01 (0.99-1.03)	0.99 (0.97-1.01)	1.05 (1.02-1.07)
High	33.1	1.00	1.00	1.00	1.00	1.00	1.00

Men	N = 49368						

	weighted %						
Occupational prestige							
Low	30.4	3.61 (3.52-3.70)	3.56 (3.47-3.64)	3.08 (3.01-3.16)	1.55 (1.50-1.60)	0.97 (0.94-1.01)	1.00 (0.96-1.05)
Medium	38.9	2.15 (2.10-2.21)	2.15 (2.10-2.20)	2.05 (2.00-2.11)	1.21 (1.17-1.25)	0.88 (0.85-0.91)	0.92 (0.89-0.95)
High	30.7	1.00	1.00	1.00	1.00	1.00	1.00

Model I, additionally adjusting for survey year and marital status; Model II, additionally adjusting for previous sickness; Model III, additionally adjusting for education and income; Model IV, additionally adjusting for occupational class; Model V, additionally adjusting for employment type, contract type, and employment sector
