# Supplementary material for: Occupational prestige and sickness absence inequality in employed women and men in Sweden: a registry-based study
Source: BMJ Open. 2021 Jun 8;11(6):e050191. doi: 10.1136/bmjopen-2021-050191 (PMC8190050; doi:10.1136/bmjopen-2021-050191)
Supplement: Supplementary data [file bmjopen-2021-050191supp002.pdf]

Online supplementary table 2. Association of occupational prestige with long-term sickness absence among those with at least one spell of sickness absence. Odds ratios (OR) and 95% confidence intervals (95%CI) obtained from generalized estimating equation (GEE) with logistic regression

	Total N = 21836	Age-adjusted	Model I	Model II	Model III	Model IV	Model V
	weighted %	OR (95%CI)	OR (95%CI)	OR (95%CI)	OR (95%CI)	OR (95% CI)	OR (95% CI)
Occupational prestige							
Low	43.4	1.51 (1.48-1.54)	1.51 (1.48-1.54)	1.38 (1.36-1.41)	1.03 (1.00-1.05)	1.08 (1.04-1.11)	1.07 (1.04-1.11)
Medium	33.4	1.11 (1.08-1.13)	1.12 (1.09-1.14)	1.07 (1.04-1.09)	0.91 (0.89-0.93)	0.98 (0.96-1.01)	1.00 (0.98-1.03)
High	23.2	1.00	1.00	1.00	1.00	1.00	1.00

Model I, additionally adjusting for survey year, gender, and marital status; Model II, additionally adjusting for previous sickness; Model III, additionally adjusting for education and income; Model IV, additionally adjusting for occupational class; Model V, additionally adjusting for employment type, contract type, and employment sector
