# Supplementary material for: Occupational prestige and sickness absence inequality in employed women and men in Sweden: a registry-based study
Source: BMJ Open. 2021 Jun 8;11(6):e050191. doi: 10.1136/bmjopen-2021-050191 (PMC8190050; doi:10.1136/bmjopen-2021-050191)
Supplement: Supplementary data [file bmjopen-2021-050191supp003.pdf]

Online supplementary table A3. Intersections of gender and occupational prestige and the association with long-term sickness absence among those with at least one spell of sickness absence. Odds ratios (OR) and 95% confidence intervals (95%CI) obtained from generalized estimating equation (GEE) with logistic regression

	Total N = 21836	Age-adjusted	Model I	Model II	Model III	Model IV	Model V
	weighted %	OR (95%CI)	OR (95%CI)	OR (95% CI)	OR (95% CI)	OR (95%CI)	OR (95%CI)
Women/low prestige occup.	26.7	2.09 (2.01-2.16)	2.08 (2.00-2.15)	1.70 (1.64-1.77)	1.05 (1.00-1.09)	1.11 (1.06-1.16)	1.05 (1.00-1.11)
Women/medium prestige occup.	18.1	1.53 (1.47-1.59)	1.51 (1.46-1.57)	1.30 (1.25-1.35)	0.93 (0.89-0.96)	1.02 (0.98-1.06)	1.02 (0.98-1.06)
Women/high prestige occup.	16.3	1.49 (1.43-1.55)	1.49 (1.44-1.55)	1.32 (1.27-1.37)	1.08 (1.04-1.12)	1.09 (1.05-1.14)	1.06 (1.02-1.11)
Men/low prestige occup.	16.7	1.92 (1.86-2.00)	1.87 (1.80-1.94)	1.67 (1.61-1.73)	1.22 (1.17-1.27)	1.30 (1.24-1.37)	1.31 (1.25-1.38)
Men/medium prestige occup.	15.3	1.43 (1.37-1.87)	1.41 (1.35-1.46)	1.30 (1.25-1.35)	1.08 (1.03-1.12)	1.16 (1.11-1.21)	1.19 (1.13-1.24)
Men/high prestige occup.	6.8	1.00	1.00	1.00	1.00	1.00	1.00

Model I, additionally adjusting for survey year and marital status; Model II, additionally adjusting for previous sickness; Model III, additionally adjusting for education and income; Model IV, additionally adjusting for occupational class; Model V, additionally adjusting for employment type, contract type, and employment sector
