# Supplementary material for: Occupational prestige and sickness absence inequality in employed women and men in Sweden: a registry-based study
Source: BMJ Open. 2021 Jun 8;11(6):e050191. doi: 10.1136/bmjopen-2021-050191 (PMC8190050; doi:10.1136/bmjopen-2021-050191)
Supplement: Author's manuscript [file bmjopen-2021-050191.draft_revisions.pdf]

BMJ Open

BMJ Open is committed to open peer review. As part of this commitment we make the peer review history of every article we publish publicly available.

When an article is published we post the peer reviewers' comments and the authors' responses online. We also post the versions of the paper that were used during peer review. These are the versions that the peer review comments apply to.

The versions of the paper that follow are the versions that were submitted during the peer review process. They are not the versions of record or the final published versions. They should not be cited or distributed as the published version of this manuscript.

BMJ Open is an open access journal and the full, final, typeset and author-corrected version of record of the manuscript is available on our site with no access controls, subscription charges or pay-per-view fees (http://bmjopen.bmj.com).

If you have any questions on BMJ Open's open peer review process please email info.bmjopen@bmj.com

BMJ Open

# Occupational prestige and sickness absence inequality in employed women and men in Sweden: a registry-based study



| | |
|---|---|
| Journal: | *BMJ Open* |
| Manuscript ID | bmjopen-2021-050191 |
| Article Type: | Original research |
| Date Submitted by the Author: | 13-Feb-2021 |



| | |
|---|---|
| Complete List of Authors: | Nwaru, Chioma; University of Gothenburg, School of Public Health and Community Medicine, Institute of Medicine, Sahlgrenska Academy<br>Berglund, Tomas; University of Gothenburg, Department of Sociology and Work Science<br>Hensing, Gunnel; University of Gothenburg, School of Public Health and Community Medicine, Institute of Medicine, Sahlgrenska Academy |



| | |
|---|---|
| Keywords: | SOCIAL MEDICINE, EPIDEMIOLOGY, OCCUPATIONAL & INDUSTRIAL MEDICINE |
| | |


SCHOLARONE™
Manuscripts

*I, the Submitting Author has the right to grant and does grant on behalf of all authors of the Work (as defined in the below author licence), an exclusive licence and/or a non-exclusive licence for contributions from authors who are: i) UK Crown employees; ii) where BMJ has agreed a CC-BY licence shall apply, and/or iii) in accordance with the terms applicable for US Federal Government officers or employees acting as part of their official duties; on a worldwide, perpetual, irrevocable, royalty-free basis to BMJ Publishing Group Ltd ("BMJ") its licensees and where the relevant Journal is co-owned by BMJ to the co-owners of the Journal, to publish the Work in this journal and any other BMJ products and to exploit all rights, as set out in our licence.*

*The Submitting Author accepts and understands that any supply made under these terms is made by BMJ to the Submitting Author unless you are acting as an employee on behalf of your employer or a postgraduate student of an affiliated institution which is paying any applicable article publishing charge ("APC") for Open Access articles. Where the Submitting Author wishes to make the Work available on an Open Access basis (and intends to pay the relevant APC), the terms of reuse of such Open Access shall be governed by a Creative Commons licence – details of these licences and which Creative Commons licence will apply to this Work are set out in our licence referred to above.*

*Other than as permitted in any relevant BMJ Author's Self Archiving Policies, I confirm this Work has not been accepted for publication elsewhere, is not being considered for publication elsewhere and does not duplicate material already published. I confirm all authors consent to publication of this Work and authorise the granting of this licence.*

For peer review only

# Occupational prestige and sickness absence inequality in employed women and men in Sweden: a registry-based study

Chioma A. Nwaru,[1] Tomas Berglund,[2] Gunnel Hensing[1]

[1]School of Public Health and Community Medicine, Institute of Medicine, Sahlgrenska Academy, University of Gothenburg, Gothenburg, Sweden

[2]Department of Sociology and Work Science, University of Gothenburg, Gothenburg, Sweden,

**Correspondence to** Chioma A. Nwaru, School of Public Health and Community Medicine, Institute of Medicine, Sahlgrenska Academy, University of Gothenburg, Box 414, 40530, Gothenburg, Sweden; chioma.nwaru@gu.se

**Key words:** occupational prestige, gender, sickness absence

**Word count**
Abstract: 294
Main text: 3982



For peer review only - http://bmjopen.bmj.com/site/about/guidelines.xhtml

## ABSTRACT

**Objectives** To examine the association between occupational prestige and sickness absence, and the distribution of the association in women and men. To investigate the effect of intersections of gender and occupational prestige on sickness absence.

**Design** Longitudinal

**Setting** A nationwide representative sample of Swedish working population

**Participants** 97 397 employed individuals aged 25-59 years selected from the 2004, 2007 and 2010 waves of the Swedish Labour Force Survey and prospectively linked to the Swedish Longitudinal Integration Database for Health Insurance and Labour Market Studies.

**Outcome measures** The number of sickness absence days in any particular year during a 3-year follow-up and long-term (>120 days) sickness absence based on those with at least one sick leave spell during the follow-up.

**Results** Occupational prestige was weakly associated with sickness absence in the total sample after adjusting for potential confounders. In the gender-stratified analysis, women in lower occupational prestige had higher absenteeism rates than women in high prestige occupations; men in lower occupational prestige had higher odds for long-term SA than men in high prestige occupations. In the intersectional analysis, women regardless of prestige level and men in lower prestige occupations had higher probability of sickness absence compared with men in high prestige occupations. Women in high prestige occupations had the highest absenteeism rates (IRR, 2.25, 95%CI, 2.20-2.31) followed by women in medium prestige occupations (IRR, 2.01, 95%CI, 1.95-2.06), women in low prestige occupations (IRR, 1.68, 95%CI, 1.62-1.74), men in low prestige occupations (IRR, 1.37, 95%CI, 1.32-1.42), and men in medium prestige occupations (IRR, 1.17, 95%CI, 1.13-1.20). Compared with the rest of the groups, men in low and medium prestige occupations had higher odds for long-term absence.

For peer review only

**Conclusions** There is need to pay close attention to occupational prestige as a factor that may influence health and labour market participation.

For peer review only



For peer review only - http://bmjopen.bmj.com/site/about/guidelines.xhtml

**Strengths and limitations of this study**

- A large, reliable, nationally representative sample of employed women and men in Sweden was used for the study
- Data on sickness absence was register-based
- The incorporation of gender-occupational prestige intersections expanded previous knowledge on gender differences in sickness absence
- Occupational prestige was based on job titles provided in the inclusion year, which may not necessarily be the longest held occupations for some of the participants
- The study did not include sickness absence lasting 14 days or less.

For peer review only



For peer review only - http://bmjopen.bmj.com/site/about/guidelines.xhtml

# INTRODUCTION

Sickness absence (SA), particularly long-term absence, has consistently been associated with unemployment,[1] disability retirement,[2] and mortality,[3] and could therefore lead to permanent exit from the workforce. Occupation is a key indicator of socioeconomic position (SEP), and it plays an important role in determining the occurrence of SA. Compared with other SEP indicators, such as education and income, occupation is a more complex factor; it does not have a single measure that captures the occupation-health relationship.[4] In previous studies on occupation and SA, some authors classified individuals into occupational groups based on employment relations[5] and job titles.[6] Other researchers have done so by combining information on job titles, education, and similarity of work content.[7] Several other occupational classification schemes exist that have not been studied in relation to SA. Because different occupation-based measures originate from different theoretical concepts, each classification tends to capture an aspect of occupation itself as related to health. Moreover, the mechanisms connecting occupation to health are not similar across different occupation-based measurements.[4, 8] To better understand the role of various aspects of occupation on SA, which is important for creating interventions to reduce SA, an exploration of different occupation-based measures, particularly those that have received less attention, is warranted.

The present study focuses on the role of occupational prestige as a determinant of SA. Occupational prestige as used here reflects social status, given that the measure represents the value placed on different occupations by the society. Occupational prestige is measured using the Standard International Occupational Prestige Scale (SIPOS), which is a scale that provides hierarchical ranking from least to most esteemed occupations according to average societal ratings.[4, 9] No studies so far have investigated the association between occupation and SA using SIOPS-based occupational prestige as an indicator. Investigating this



For peer review only - http://bmjopen.bmj.com/site/about/guidelines.xhtml

association can help to capture features of social hierarchy (acceptance, respect, recognition, admiration, autonomy, power, social network and social support [10, 11] that may not be adequately represented in other measures of occupational classification, such as occupational class, thus illuminating how social position and its associated rewards and privileges contribute in shaping health risk.

Occupation is a major contributor of an individual's identity in society as well as an impactor on individual's mental health. The position a given occupation has on the prestige scale strongly impacts both the adoption of an occupational identity and construction of self-esteem.[12-14] Compared to individuals in lower prestige occupations, those in high prestige occupations are more likely to identify with their occupations because of the social rewards and privileges associated with high status occupations.[12, 13] Owing to a lack of such rewards and privileges, employees in lower prestige occupations may have increased risk of work alienation (i.e. psychological detachment from their jobs), low self-esteem, poor work satisfaction, and reduced social interactions.[13, 14] These negative affects can induce poor response to stress and consequently increase the risk of poor health.[15, 16] Previous research has shown that occupational prestige is a determinant of different health outcomes, and that it can influence health independently of other SEP indicators and job characteristics. For example, Fujishuro *et al.*[17] reported increased poor self-rated health among employees in lower prestige occupations after controlling for occupational categories, education, income, job stress, workplace social support, and job satisfaction. Additionally, occupational prestige was found to be a determinant of self-rated health[18] as well as a determinant of mortality,[19] even when controlling for education and income in both studies. In line with these studies, we expect to find increased risk of SA among employees working in lower prestige occupations.



For peer review only - http://bmjopen.bmj.com/site/about/guidelines.xhtml

Gender is an important characteristic in SA research. A common statistical approach when investigating gender differences in SA is to compare the risk in women and men, while controlling for potential confounders. Thus, in several studies women are treated as a homogenous group, and reported as having higher sickness absence than men.[20,21] Hankivsky et al.[22] noted that such an approach fails to recognize the diversity of women regarding other forms of social identities (for example, status), as well as the relational nature among different social identities. They suggested that researchers incorporate intersectional approach, i.e., investigate the extent to which gender combines with other social identities to create health inequality. The intersectional approach integrates both between and within group differences,[23] therefore offering the potential to provide insight on different levels of SA inequality.

Our aims in this study are to (1) investigate whether occupational prestige is associated with SA, (2) examine the distribution of this association among women and men, and (3) investigate the effects of intersections of gender and occupational prestige on SA. The current study has the potential to improve knowledge on determinants of SA as well as on target groups, both of which are crucial for reducing SA and improving labour market participation.

# METHODS

## Study population and design

This study is part of the New Ways and the Polarization programs at the University of Gothenburg. The study is based on data of participants in the Swedish Labour Force Survey (LFS), which is a quarterly survey used to monitor labour market developments for the entire population aged 15–74 years. The LFS utilizes a rotating sampling design, meaning that



For peer review only - http://bmjopen.bmj.com/site/about/guidelines.xhtml

participants in the survey are interviewed once every quarter for a maximum of eight times over a 2-year period, after which they are replaced with new sample individuals.[24] For this study, we selected from three cross-sections at the end of the survey years 2004, 2007, and 2010, individuals aged 25–59 years who were employed wage earners according to their main economic activity and were at work, i.e., not self-employed, unemployed, retired or on disability pension (N = 107 608). Using the participants' encrypted identification number, we linked information from the cohorts with the LISA (Swedish Longitudinal Integration Database for Health Insurance and Labour Market Studies) register [25] and followed each cohort for 3 years thus covering the period 2005–2013. We excluded from the cohorts those participants who started receiving old age or disability pension by the end of the study period (n = 6278). We also excluded participants with incomplete follow-up data and those with missing exposure or potential control variables (n = 3933), resulting in a final working sample of 97 397 participants.

Patient and public involvement

This study was performed without patient or public involvement. The Swedish national registers are protected by special legislation that makes it possible for researchers to collect certain information without personal consent.

Ethical approval

The regional ethical review board in Gothenburg approved the study (Dnr: 090-17). All the data used in this study were anonymised and the researchers do not have access to any personal information that could identify individuals included in the study.



For peer review only - http://bmjopen.bmj.com/site/about/guidelines.xhtml

## Measurements

Occupational prestige

We measured occupational prestige, the exposure in this study, with the SIOPS.[9] The scores, which ranged from 13 to 78, were calculated for occupations on the three-digit level in the ISCO-88 classification. This classification is similar to the Swedish Occupational Standard (SSYK-96) that was applied during 2004, 2007, and 2010. Based on the distribution of occupational prestige in the current data, we divided the scores into tertiles (15–34 points, 35–50 points, and 51–78 points) to represent low, medium and high occupational prestige categories, respectively. The last category was used as the reference in the analyses. We constructed six intersectional groups using information on the prestige groups and on gender (obtained from the LFS). The groups were women in low prestige occupations, women in medium prestige occupations, women in high prestige occupations, men in low prestige occupations, men in medium prestige occupations, and men in high prestige occupations (reference group).

Sickness absence

In Sweden, all individuals aged 16 years and above with an income from work or unemployment benefits are entitled to sickness absence benefits. If the sick leave lasts for more than seven consecutive days, starting from the eighth day, a doctor's certificate is required. During the study observation years (2005–2013), the employers paid for absence days for 14 days or less after a qualifying day without benefits. From day 15 onward, the Social Insurance Agency (SIA) pays SA benefits. Unemployed individuals have one qualifying day and receive sick pay from the SIA from the second day. In this study, information on SA was based on data from the LISA that only contains information on SA from day 15 onward. We used two measures of SA: the number of SA days in any particular year during the follow-up and long-term SA. The latter was based on those with at least one

For peer review only



For peer review only - http://bmjopen.bmj.com/site/about/guidelines.xhtml

sick leave spell during the follow-up and defined as absence days lasting over 120 days. In the so-called rehabilitation chain in Sweden, evaluation of work capacity and the right to SA benefit are assessed at specific times, starting from day 90 into the SA period. By choosing 120 days as the cut point when the individuals would have undertaken the first assessment, our measure of long-term SA is more likely to represent serious health conditions, which are of interest in this study.

Potential confounders

From the LFS database, we extracted information on participants' age (25–34, 35–44, and 45–59 years), marital status (single, cohabiting, and married), occupational class (manual, lower non-manual, and higher non-manual), employment sector (private or public), employment type (temporary or permanent), and contract type (full-time, part-time) at the time of inclusion in the study. From the LISA register, we obtained information on previous SA for the 2 years preceding the study observation period. We also retrieved information on highest education attained (primary, secondary, or tertiary) and on gross annual salaries (divided into quartiles) of the participants during the inclusion year from the LISA database. We categorized the year of measurement as 2004, 2007, and 2010. We treated these variables as confounders capable of influencing the association between occupational prestige and SA.

## Statistical analyses

Data analyses were performed with the generalized estimating equation (GEE), a statistical technique suitable for modelling repeated outcome variables. The technique takes into account the within-subject correlation of the repeated SA variable.[26] The annual rate of SA days was estimated using GEE with negative binomial. The negative binomial distribution was chosen because of the overdispersion in the count outcome variable.[27] Exchangeable correlation was specified in all the models as the correlation structure based on the result of a preliminary investigation. However, GEE is robust to misspecification of the



For peer review only

correlation structure.[26] Five separate models were estimated, starting with a crude model followed by four adjusted models. In the first adjusted model (Model I), survey year, age, gender, marital status, and previous SA were entered simultaneously into the model. In order to estimate the effect of occupational prestige on SA in the presence of other commonly used SEP indicators, additional adjustment was made for income and education in Model II, and occupational class in Model III. Employment-related characteristics, which we interpreted as proxy measures for work-related characteristics, were added in the final model (Model IV). The coefficients were expressed as incidence rate ratios (IRRs) and 95% confidence intervals (CIs).

Regarding the risk for long-term SA, GEE with logistic regression was used because the outcome was binary (≤120 versus >120 SA days). The modelling strategy was the same as for the count outcome. Because the Swedish evaluation of work capacity within the rehabilitation framework starts at day 90, the robustness of the results regarding long-term SA was tested by repeating the analyses using 90 days as the cut point. All the analyses were population weighted to correct for biases due to sampling. IBM SPSS version 26 for Windows was used for all the statistical analyses.



For peer review only - http://bmjopen.bmj.com/site/about/guidelines.xhtml

Table 1. Distribution of sample characteristics by occupational prestige at baseline and the association of the characteristics with sickness absence at follow-up

| | Total | Occupational prestige at baseline | | | | Incidence rate ratios (IRR) associated with number of SA† days at follow-up | Odds ratios (OR) associated with long-term sickness absence at follow-up |
|---|---|---|---|---|---|---|---|
| | N = 97397 | | | | | N = 97397 | N = 21836† |
| | | Low (n = 32343) | Medium (n = 34556) | High (n = 30498) | p-value | | |
| | n (weighted %) | Weighted % | Weighted % | Weighted % | | IRR (95%CI) | OR (95%CI) |
| Survey year | | | | | 0.000 | | |

For peer review only

| | | | | | | | |
|---|---|---|---|---|---|---|---|
| 2004 | 27950 (34.1) | 32.2 | 36.7 | 31.0 | | 1.32 (1.31-1.34) | 1.48 (1.45-1.50) |
| 2007 | 31483 (32.8) | 32.8 | 35.3 | 31.9 | | 0.88 (0.87-0.89) | 0.97 (0.96-0.99) |
| 2010 | 37964 (33.1) | 32.5 | 34.9 | 32.6 | | 1.00 | 1.00 |
| Age (years) | | | | | 0.000 | | |
| 25-34 | 26259 (27.7) | 34.2 | 35.3 | 30.5 | | 1.00 | 1.00 |
| 35-44 | 30890 (32.1) | 31.0 | 36.2 | 32.8 | | 1.32 (1.30-1.34) | 1.38 (1.35-1.41) |
| 45-59 | 40248 (40.2) | 32.5 | 35.5 | 32.0 | | 1.63 (1.61-1.65) | 1.49 (1.46-1.51) |
| Gender | | | | | 0.000 | | |
| Female | 48029 (47.5) | 34.8 | 32.1 | 33.1 | | 1.87 (1.85-1.89) | 1.11 (1.09-1.13) |
| Male | 49368 (52.5) | 30.4 | 38.9 | 30.7 | | 1.00 | 1.00 |
| Education | | | | | 0.000 | | |
| Primary | 9668 (10.0) | 59.5 | 33.5 | 7.0 | | 2.16 (2.13-2.20) | 1.56 (1.53-1.59) |
| Secondary | 46415 (47.2) | 47.2 | 39.7 | 13.1 | | 1.57 (1.55-1.59) | 1.21 (1.19-1.23) |
| Tertiary | 41314 (42.7) | 10.0 | 31.7 | 58.4 | | 1.00 | 1.00 |
| Occupational class | | | | | 0.000 | | |
| Manual | 40389 (40.8) | 73.7 | 26.2 | 0.2 | | 1.00 | 1.00 |
| Lower non-manual | 38522 (39.5) | 6.0 | 61.2 | 32.8 | | 0.58 (0.57-0.58) | 0.75 (0.73-0.76) |



For peer review only - http://bmjopen.bmj.com/site/about/guidelines.xhtml

For peer review only

| | | | | | | | |
|---|---|---|---|---|---|---|---|
| Higher non-manual | 18486 (19.7) | 0.1 | 4.2 | 95.7 | | 0.38 (0.37-0.38) | 0.70 (0.69-0.72) |
| Marital status | | | | | 0.000 | | |
| Single | 23441 (24.3) | 37.2 | 34.8 | 28.0 | | 1.25 (1.24-1.26) | 1.29 (1.27-1.31) |
| Cohabiting | 28386 (28.9) | 33.7 | 37.6 | 28.7 | | 0.93 (0.92-0.94) | 0.95 (0.93-0.96) |
| Married | 45570 (46.8) | 29.3 | 34.9 | 35.8 | | 1.00 | 1.00 |
| Individual income | | | | | 0.000 | | |
| Lowest | 24365 (25.2) | 54.3 | 29.5 | 16.2 | | 5.63 (5.54-5.71) | 3.40 (3.31-3.49) |
| Second quartile | 24369 (24.8) | 41.0 | 38.7 | 20.3 | | 3.12 (3.07-3.17) | 1.92 (1.87-1.97) |
| Third quartile | 24330 (24.5) | 26.1 | 42.1 | 31.9 | | 2.18 (2.15-2.22) | 1.58 (1.53-1.62) |
| Highest | 24333 (25.5) | 8.9 | 32.6 | 58.5 | | 1.00 | 1.00 |
| Employment sector | | | | | 0.000 | | |
| Private | 63136 (66.5) | 31.9 | 41.5 | 26.6 | | 1.00 | 1.00 |
| Public | 34261 (33.5) | 33.7 | 24.0 | 42.3 | | 1.65 (1.63-1.67) | 1.27 (1.25-1.29) |
| Employment type | | | | | 0.000 | | |
| Temporary | 8764 (9.1) | 41.5 | 25.9 | 32.6 | | 1.14 (1.12-1.16) | 1.17 (1.15-1.20) |
| Permanent | 88633 (90.9) | 31.6 | 36.6 | 31.8 | | 1.00 | 1.00 |
| Contract type | | | | | 0.000 | | |



For peer review only - http://bmjopen.bmj.com/site/about/guidelines.xhtml

| | | | | | | | |
|---|---|---|---|---|---|---|---|
| Part-time | 19641 (19.3) | 49.6 | 27.4 | 23.0 | | 1.60 (1.58-1.61) | 1.33 (1.31-1.35) |
| Full-time | 77756 (80.7) | 28.4 | 37.6 | 34.0 | | 1.00 | 1.00 |
| Previous SA | | | | | 0.000 | | |
| No | 81995 (84.8) | 30.4 | 36.1 | 33.6 | | 1.00 | 1.00 |
| Yes | 15402 (15.2) | 44.4 | 33.4 | 22.3 | | 5.04 (4.99-5.09) | 2.45 (2.42-2.49) |

† Analyses performed among those with at least one spell of registered sickness absence during follow-up

For peer review only

# RESULTS

## Descriptive statistics

The mean age of the study population was 42 years (SD, 9.7 years). The participants' characteristics, overall and by occupational prestige groups, are presented in table 1. There were more women (than men) in low prestige occupations; the same applied for temporary (versus permanent) and part-time (versus full-time) employees, and those with previous SA (versus those without). Working in high prestige occupations was prevalent among those in higher non-manual occupations, those with a tertiary education, and those in the highest income quartiles. During the study follow-up, 22% (21 836) of the participants had at least one episode of SA lasting more than 14 days. Sixteen percent (n = 3709) of the 21 836 had at least one episode of SA lasting over 120 days. All participants' characteristics were associated with both the number of SA days and long-term absence at follow-up (table 1).

## Occupational prestige differences in number of sickness absence days

Table 2 shows the IRRs for the association between occupational prestige and number of SA days in the total sample. There was a graded association between occupational prestige and SA in the crude model. Employees in low prestige occupations had over two times more SA than those in high prestige group. Those in medium prestige occupations had 1.39 times more SA than employees in high prestige group. Adjustment for survey year, age, gender, marital status and previous SA slightly attenuated the IRR for those in low occupational prestige groups and increased the IRR for employees in medium occupational prestige group. Adjustment for income and education attenuated the IRRs for both groups. Adjustment for occupational class eliminated the increased IRR among employees in lower occupational



For peer review only - http://bmjopen.bmj.com/site/about/guidelines.xhtml

prestige. Adjustment for employment-related variables did not seem to have much influence on the association.

When the data were analysed separately for women and men, the result from the crude model (online supplementary table A1) showed significant occupational prestige differences in both sexes. As with the total sample, adjusting for income and education (Model II) and occupational class (Model III) significantly reduced the estimates. After adjusting for all potential confounders, among women, the IRR remained statistically significant among women in low (IRR, 1.22; 95% CI, 1.18–1.27) and medium (IRR, 1.05; 95% CI, 1.02–1.07) occupational prestige groups compared with women in the high occupational prestige group. Among men, no increased IRR was found both for men in low (IRR, 1.00; 95% CI, 0.96–1.05) and men in medium (IRR, 0.92; 95% CI, 0.89–0.95) prestige occupations compared with men in high prestige occupations.

Table 3 presents the distribution of intersectional groups and the IRR across the groups, with men in the high occupational prestige group as the reference group. All the groups had increased absence rates compared with men in high prestige occupations in the crude model. After accounting for all potential confounders, women in high prestige occupations had the highest absence rates (IRR, 2.25, 95% CI, 2.20–2.31), which is an unexpected finding in this study. Women in medium prestige occupations had the second highest absence rate (IRR, 2.01, 95% CI, 01.95–2.06) followed by women in low prestige occupations (IRR, 1.68, 95% CI, 1.62-1.74), men in low prestige occupations (IRR, 1.37, 95% CI, 1.32-1.42), and men in medium prestige occupations (IRR, 1.17, 95% CI, 1.13-1.20).



For peer review only - http://bmjopen.bmj.com/site/about/guidelines.xhtml

Table 2. Association of occupational prestige with number of sickness absence days at follow-up. Incidence rate ratios (IRR) and 95% confidence intervals (95%CI) obtained from generalized estimating equation (GEE) with negative binomial regression

| | Total N = 97397 n (weighted %) | Crude IRR (95%CI) | Model I IRR (95%CI) | Model II IRR (95%CI) | Model III OR (95%CI) | Model IV OR (95% CI) |
|---|---|---|---|---|---|---|
| Occupational prestige | | | | | | |
| Low | 32342 (32.5) | 2.45 (2.42-2.48) | 2.35 (2.32-2.38) | 1.44 (1.41-1.47) | 1.01 (0.98-1.04) | 1.02 (1.00-1.05) |
| Medium | 34556 (35.7) | 1.39 (1.38-1.41) | 1.53 (1.50-1.55) | 1.12 (1.11-1.14) | 0.96 (0.94-0.98) | 1.00 (0.98-1.02) |
| High | 30498 (31.8) | 1.00 | 1.00 | 1.00 | 1.00 | 1.00 |

Model I adjusted for survey year, age, gender, marital status, and previous sickness; Model II, additionally adjusting for education and income; Model III additionally adjusting for occupational class; Model IV additionally adjusting for employment type, contract type, and employment sector

For peer review only



For peer review only - http://bmjopen.bmj.com/site/about/guidelines.xhtml

Table 3. Intersections of gender and occupational prestige and the association with number of sickness absence days at follow-up. Incidence rate ratios (IRR) and 95% confidence intervals (95%CI) obtained from generalized estimating equation (GEE) with negative binomial regression

| | Total N = 97397 n (weighted %) | Crude IRR (95%CI) | Model I IRR (95%CI) | Model II IRR (95%CI) | Model III IRR (95%CI) | Model IV IRR (95%CI) |
|---|---|---|---|---|---|---|
| Female/low occupational prestige | 17150 (16.5) | 5.92 (5.78-6.05) | 4.91 (4.80-5.02) | 2.56 (2.49-2.64) | 1.76 (1.70-1.82) | 1.68 (1.62-1.74) |
| Female/medium occupational prestige | 15284 (15.2) | 3.48 (3.40-3.57) | 3.00 (2.93-3.08) | 2.11 (2.06-2.17) | 1.98 (1.93-2.03) | 2.01 (1.95-2.06) |
| Female/high occupational prestige | 15595 (15.7) | 2.91 (2.84-2.98) | 2.68 (2.61-2.74) | 2.38 (2.32-2.43) | 2.35 (2.30-2.41) | 2.25 (2.20-2.31) |
| Male/low occupational prestige | 15193 (16.0) | 3.56 (3.47-3.64) | 3.11 (3.04-3.19) | 1.93 (1.88-1.99) | 1.34 (1.29-1.38) | 1.37 (1.32-1.42) |
| Male/medium occupational prestige | 19272 (20.4) | 2.13 (2.08-2.18) | 2.06 (2.01-2.11) | 1.43 (1.39-1.47) | 1.12 (1.09-1.15) | 1.17 (1.13-1.20) |
| Male/high occupational prestige | 14903 (16.1) | 1.00 | 1.00 | 1.00 | 1.00 | 1.00 |

Model I adjusted for survey year, age, marital status, and previous sickness; Model II, additionally adjusting for education and income; Model III additionally adjusting for occupational class; Model IV additionally adjusting for employment type, contract type, and employment sector



For peer review only - http://bmjopen.bmj.com/site/about/guidelines.xhtml

## Occupational prestige differences in long-term sickness absence

Table 4 shows the ORs for the association between occupational prestige and long-term SA in the total sample. After adjustment for all potential confounders, only the OR for employees in low prestige occupations showed slightly increased OR for long-term SA. In the gender-stratified results seen in table 5, among men, there was a strong graded association between occupational prestige and long-term SA in the crude model. Adjustment for survey year, age, marital status, previous sickness absence (Model I), as well as education and income (Model II) had an attenuating effect, while adjustment for occupational class (Model III) increased the ORs. After accounting for all potential confounders, men in low and medium prestige occupations had 26% and 14% increased odds, respectively, compared with men in high prestige occupations (Model IV). Among women, there were no occupational prestige differences and no increased ORs with respect to long-term SA after accounting for all potential confounders (Model IV).

The results with the intersectional groups showed crude ORs ranging from 1.41 to 2.03, with the highest OR found among women in low prestige occupations (online supplementary table A2). However, in the full model (Model IV), men in low prestige occupations had the highest risk (OR, 1.31, 95% CI, 1.25-1.38), followed by men in medium prestige occupations (OR, 1.19, 95% CI, 1.13-1.24), women in high prestige occupations (OR, 1.06, 95% CI, 1.02-1.11), and women in low prestige occupations (OR, 1.05, 95% CI, 01.00-1.11). No statistically significant OR was found among women in medium prestige occupations (OR, 1.02, 95%CI, 0.98-1.06). The estimates pointed toward the same direction when we repeated the analyses using 90 days as the cut point (data not shown).



For peer review only - http://bmjopen.bmj.com/site/about/guidelines.xhtml

Table 4. Association of occupational prestige with long-term sickness absence among those with at least one spell of sickness absence. Odds ratios (OR) and 95% confidence intervals (95%CI) obtained from generalized estimating equation (GEE) with logistic regression

| | Total N = 21836 n (weighted %) | Crude OR (95%CI) | Model I OR (95%CI) | Model II OR (95%CI) | Model III OR (95%CI) | Model IV OR (95% CI) |
|---|---|---|---|---|---|---|
| Occupational prestige | | | | | | |
| Low | 9564 (43.4) | 1.51 (1.49-1.54) | 1.38 (1.36-1.41) | 1.03 (1.00-1.05) | 1.08 (1.04-1.11) | 1.07 (1.04-1.11) |
| Medium | 7256 (33.4) | 1.11 (1.09-1.13) | 1.07 (1.04-1.09) | 0.91 (0.89-0.93) | 0.98 (0.96-1.01) | 1.00 (0.98-1.03) |
| High | 5016 (23.2) | 1.00 | 1.00 | 1.00 | 1.00 | 1.00 |

Model I adjusted for survey year, age, gender, marital status, and previous sickness; Model II, additionally adjusting for education and income; Model III additionally adjusting for occupational class; Model IV additionally adjusting for employment type, contract type, and employment sector



For peer review only - http://bmjopen.bmj.com/site/about/guidelines.xhtml

Table 5. Gender-stratified association between occupational prestige and long-term sickness absence among those with at least one spell of sickness absence.

Odds ratios (OR) and 95% confidence intervals (95%CI) obtained from generalized estimating equation (GEE) with logistic regression

| | | Crude<br>OR (95%CI) | Model I<br>OR (95%CI) | Model II<br>OR (95%CI) | Model III<br>OR (95%CI) | Model IV<br>OR (95%CI) |
|---|---|---|---|---|---|---|
| Female | N = 13719<br>n (weighted %) | | | | | |
| Occupational prestige | | | | | | |
| Low | 6098 (43.7) | 1.41 (1.38-1.44) | 1.30 (1.27-1.33) | 1.06 (1.03-1.09) | 1.01 (0.96-1.05) | 0.98 (0.94-1.03) |
| Medium | 4020 (29.6) | 1.03 (1.00-1.05) | 0.99 (0.96-1.01) | 0.90 (0.87-0.92) | 0.95 (0.93-0.98) | 0.98 (0.95-1.01) |
| High | 3601 (26.7) | 1.00 | 1.00 | 1.00 | 1.00 | 1.00 |
| Male | N = 8117<br>n (weighted %) | | | | | |
| Occupational prestige | | | | | | |
| Low | 3466 (43.0) | 1.89 (1.82-1.96) | 1.64 (1.58-1.71) | 1.07 (1.02-1.12) | 1.26 (1.19-1.34) | 1.26 (1.19-1.34) |
| Medium | 3236 (39.4) | 1.41 (1.36-1.47) | 1.28 (1.23-1.33) | 0.97 (0.92-1.01) | 1.12 (1.06-1.19) | 1.14 (1.08-1.21) |
| High | 1415 (17.6) | 1.00 | 1.00 | 1.00 | 1.00 | 1.00 |



For peer review only - http://bmjopen.bmj.com/site/about/guidelines.xhtml

Model I adjusted for survey year, age, marital status, and previous sickness; Model II, additionally adjusting for education and income; Model III additionally adjusting for occupational class; Model IV additionally adjusting for employment type, contract type, and employment sector

For peer review only



For peer review only - http://bmjopen.bmj.com/site/about/guidelines.xhtml

# DISCUSSION

We found a weak association between occupational prestige and SA in the total sample after controlling for survey, age, gender, marital status, education, income, occupational class and employment-related variables. However, when we assessed the distribution of the association separately in women and men, we found a graded association between occupational prestige and SA in both sexes. Women in lower prestige occupations had higher rates of SA days than women in high prestige occupations. Men in lower prestige occupations had higher odds for long-term SA than men in high prestige occupations. When we pooled the sample and investigated the effect of intersections of gender and occupational prestige on SA, we found that men in high prestige occupations had the lowest IRR for SA days, as well as the lowest OR for long-term SA than the rest of the groups. Of all the groups, women in high prestige occupations had the highest IRR for SA days, while men in low prestige occupations had the highest odds for long-term SA.

Our finding of increased SA among women and men in lower occupational prestige corroborates previous studies that used different health outcomes.[17-19] Occupational class, income, and education significantly impacted on the effect estimates, but the association remained statistically significant, thus supporting the notion that occupational prestige taps into aspects of the occupation-health relationship that are not represented by education, income, and occupational class.[17] Work stress has been hypothesized as one mechanism connecting lower occupational prestige and poor health outcomes. Hoven *et al.*[28] reported that employees in lower prestige occupations had increased work stress, i.e., effort-reward imbalance, which in turn, increased their risk of depressive symptoms. Working in lower prestige occupations may also induce feelings of low self-esteem[13, 17] that may lead to increased risk of anxiety, poor social functioning, risky behaviour,[29] and poor recovery



For peer review only - http://bmjopen.bmj.com/site/about/guidelines.xhtml

from illness.[30] Mattews *et al.*[31] found that employees in lower prestige occupations more often experienced interpersonal conflicts, boredom, and increased heart rate; and interpersonal conflicts at work have been associated with increased risk of prolonged fatigue and poor general health.[32] This provides another possible reason for the increased SA among employees in lower prestige occupations.

We found increased IRR for SA days across all the intersectional groups compared with men in high prestige occupations, which was expected. Contrary to expectations, the IRR for SA days was higher for women in high prestige occupations than for women and men in lower prestige occupations. This finding supports the notion that some people occupy intersectional locations that include both privileges and marginalization.[33] The finding highlights the importance of the intersectional approach and the limitations associated with taking a unitary approach in evaluating health inequality.[34] The increased absence rate among women in high prestige occupations could reflect stressful conditions emanating from the combination of household chores and high workload. Berntsson *et al.*[35] found that women in high positions in Sweden are still mainly responsible for household tasks and childcare duties, and they reported higher levels of stressful conditions than men in similar positions.

In this study, among men, we found substantial occupational prestige differences in long-term SA, whereas among women, there was no such differences. In the intersectional analysis, men in lower prestige occupations also had higher odds for long-term SA than women regardless of prestige levels. Higher SA incidence in women than in men has been well supported in previous studies [20, 21] but longer absence spells among women has been a controversial topic in the literature, including in studies with similar cut-off points. For instance, Lidwall and Marklund[36] reported a higher risk for more than 60 absence days among women compared with men, whereas Laaksonen *et al.*[37] did not find increased



For peer review only - http://bmjopen.bmj.com/site/about/guidelines.xhtml

female absence using a cut-off point of 60 days. In Sweden[38] and abroad,[39] longer absence duration has been reported among men compared with women, which aligns with our finding of increased risk among men in lower prestige occupations. A possible explanation for this finding may relate to men's value system. Men attribute significant importance to values, such as power, status, and achievement.[40] Because they are sensitive to being relegated to a subordinate position, men in lower status occupations may develop negative perceptions of themselves, and to compensate, may engage in unhealthy behaviours that may jeopardize their psychological health.[41] Peterson[42] found that how men perceived their level of control, authority, and status had a significant impact on their psychological wellbeing, and psychiatric illness has been reported previously as a determinant of long-term SA among men in Sweden.[38] Research has also shown that men are more likely to dismiss their health needs and delay seeking health care compared with women;[43] such behaviours may worsen health situations and lead to long-term SA. The tendency of women to be more proactive in consulting professional help, may, on the one hand, explain their increased absenteeism rates, and, on the other hand, provide an alternative explanation for their decreased risk for long-term SA as observed in the current study.

The strengths of the current study include the longitudinal design, the use of register-based SA data, and the relatively long follow-up (observation period covering 2005–2013), which spanned periods of varying economic situations. The use of GEE made it possible to account for within-individual correlation in the SA variable, which is not attainable using ordinary regression methods. The application of the intersectional approach can be considered as an extension of previous studies on gender differences in SA, because it provided deeper insights on how gender and occupational prestige are mutually constituted to shape SA inequality in the working population.[23] This study was based on data of a large, reliable nationally representative sample (>70% LFS response rate) of employed women and



For peer review only - http://bmjopen.bmj.com/site/about/guidelines.xhtml

men, making it possible for the findings to be generalized to the Swedish working population. One limitation of the study relates to our measure of occupational prestige. The measure was based on job titles provided in the inclusion year, which may not necessarily be the longest held occupations for some of the participants. We could not assess the effect of some important work-related variables; for example, work demands, control, and support, which have been shown to be associated with occupational prestige,[28] and SA.[44] However, we did include several employment-related factors (contract type, employment sector, and employment type) as proxy measures for these psychosocial work characteristics, and our findings seem not to be influenced by these factors. Our measure of SA did not include absence spells lasting 14 days or less. We also did not examine cause-specific SA. It is possible that occupational prestige will exert varying effects on these forms of SA.

# CONCLUSIONS

This research demonstrated an association between lower occupational prestige and increased SA in women and men. Compared with men in high prestige occupations, women in all prestige levels and men in the lower prestige occupations had higher probability of SA days, with the highest IRR found among women in high prestige occupations. Men in low and medium prestige occupations had higher odds for long-term SA than the rest of the groups. The findings highlight the importance of expanding discourses on SA inequality to include aspects of occupation that are rarely discussed, such as occupational prestige. Interventions that would help employees in low prestige occupations to valorise their occupations may be helpful in promoting health and wellbeing and reducing SA, especially among men.



For peer review only - http://bmjopen.bmj.com/site/about/guidelines.xhtml

**Acknowledgement:** We thank Ylva Ulfsdotter Erikson for her constructive comments on this study.

**Funding:** This work was supported by [Forte] grant number [2016-07204].

**Competing interest**: None declared

**Author contributions:** CAN designed the study and was chiefly responsible for analysing the data and writing the manuscript drafts. GH participated in developing the research design, interpreting the results and writing the manuscript. TB delivered the data and contributed to the interpretation of the results and writing of the manuscript. All authors read and approved the final manuscript.

**Data availability statement**: The authors do not have the permission to share the data that support the findings of this study due to data protection regulations. Deidentified participant data are available from Statistics Sweden (scb@scb.se) on reasonable request.

# REFERENCES

1. Hultin H, Lindholm C, Möller J. Is there an association between long-term sick leave and disability pension and unemployment beyond the effect of health status?- a cohort study. PloS One 2012;7(4):e35614. Doi: 10.1371/journal.pone.0035614.
2. Salonen L, Blomgren J, Laaksonen M, Niemelä M. Sickness absence as a predictor of disability retirement in different occupational classes: a register-based study of a working-age cohort in Finland in 2007-2014. BMJ Open 2018;8(5):e020491 doi:10.1136/bmjopen-2017-020491
3. Kivimäki M, Head J, Ferrie JE, Shipley MJ, Vahtera J, Marmort MG. Sickness absence as a global measure of health: evidence from mortality in the Whitehall II prospective cohort study. BMJ 2003;327(7411):364. Doi:10.1136/bmj.327.7411.364



For peer review only - http://bmjopen.bmj.com/site/about/guidelines.xhtml

4. Connelly R, Gayle V, Lambert PS. A review of occupation-based social classifications for social survey research. Methodological Innovations 2016;9:1-14
5. Hansen H-T, Ingebrigtsen T. Social class and sickness absence in Norway. Acta Sociologica 2008;51(4)309-327
6. Piha K, Laaksonen M, Martikainen P, Rahkonen O, Lahelma E. Socio-economic and occupational determinants of work injury absence. European Journal of Public Health 2013;23(4):693-698
7. Kristensen TR, Jensen SM, Kreiner S, Mikkelsen S. Socioeconomic status and duration and pattern of sickness absence. A 1-year follow-up study of 2331 hospital employees. BMC Public Health 2010;10:643 http://www.biomecentral.com/1471-2458/10/643
8. Lambert PS, Bihagen E. Using occupation-based social classifications. *Work Employ Soc* 2014;28(3):481–94.
9. Treiman D. Occupational prestige in comparative perspective. New York: Wiley; 1977
10. Galobardes B, Shaw M, Lawlor DA, Lynch JW, Smith GD. Indicators of socioeconomic position (part 2). J Epidemiol Community Health 2006;60:95-101
11. Van Kleef GA, Cheng JT. Power, status, and hierarchy: current trends and future challenges. Current Opinion in Psychology 2020, 33: v-xiv. https://doi.org/10.1016/j.copsyc.2020.03.011
12. Ulfsdotter Eriksson Y, Linde M. "“Being” or “Doing” a profession: work as a matter of social identity”. The international Journal of Interdisciplinary Cultural Studies. 2014;8(1):33-43
13. Duemmler K, Caprani I. Identity strategies in light of a low prestige occupation: the case of retail apprentices. *J Educ Work* 2017;30(4):339–52.
14. Duemmler K, Caprani I, Felder A. The challenge of occupational prestige for occupational identities: comparing bricklaying and automation technology apprentices



For peer review only - http://bmjopen.bmj.com/site/about/guidelines.xhtml

in Switzerland. Vocations and Learning 2020;13(3):369-388. Doi: 10.1007/s12186-020-09243-3

15. Marmot MG. Status syndrome a challenge to Medicine. *JAMA* 2006;295(11):1304–7.

16. Fournier MA. Dimensions of human hierarchy as determinants of health and happiness. Current Opinion in Psychology 2020;33:110-114

17. Fujishiro K, Xu J, Gong F. What does "occupation" represent as an indicator of socioeconomic status? Exploring occupational prestige and health. *Soc Sci Med* 2010;71:2100–7.

18. Halleröd B, Gustafsson J-E. A longitudinal analysis of the relationship between changes in socio-economic status and changes in health. *Soc Sci Med* 2011;72:116–23.

19. Christ SL, Fleming LE, Lee DJ, et al. The effects of a psychosocial dimension of socioeconomic position on survival: occupational prestige and mortality among US working adults. *Sociol Health Illn* 2012;34(7):1103–17.

20. Casini A, Godin I, Clays E, Kittel F. Gender difference in sickness absence from work: a multiple mediation analysis of psychosocial factors. European Journal of Public Health 201;23(4):635-642

21. Østby KA, Mykletun A, Nilsen W. Explaining the gender gap in sickness absence. Occupational Medicine 2018;68:320-326.

22. Hankivsky O, Reid C, Cormier R, et al. Exploring the promises of intersectionality for advancing women's health research. *Int J Equity Health* 2010;9:5. doi:10.1186/1475-9276-9-5



For peer review only - http://bmjopen.bmj.com/site/about/guidelines.xhtml

23. Green MA, Evans CR, Subramanian SV. Can intersectionality theory enrich population health research? *Soc Sci Med* 2017;178:214–16. doi:10.1016/j.socscimed.2017.02.029

24. Skans LH. Labor market development according to different statistical sources. *Economic Commentaries* 2019;6:1–12.

25. Ludvigsson JF, Svedberg P, Olén O, et al. The longitudinal integrated database for health insurance and labor market studies (LISA) and its use in medical research. *Eur J Epidemiol* 2019;34:423–37. doi:10.1007/s10654-019-00511-8

26. Liang K-Y, Zeger SL. Longitudinal data analysis using generalized linear models. *Biometrika* 1986;73:13–22. doi:10.1093/biomet/73.1.13

27. Ballinger GA. Using generalized estimating equations for longitudinal data analysis. *Organ Res Methods* 2004;7(2):127–50. doi:10.1177/1094428104263672

28. Hoven H, Wahrendorf M, Siegrist J. Occupational position, work stress and depressive symptoms: a pathway analysis of longitudinal SHARE data. *J Epidemiol Community Health* 2015;69:447–52. doi:10.1136/jech-2014-205206

29. Mann M, Hosman CMH, Schealma HP, et al. Self-esteem in a broad-spectrum approach for mental health promotion. *Health Educ Res* 2004;19(4):357–72. doi:10.1093/her/cyg041

30. Yanos PT. The impact of illness identity on recovery from severe mental illness. *Am J Psychiatr Rehabil* 2010;13(2):73–93. doi:10.1080/15487761003756860

31. Matthews KA, Räikkönen K, Everson SA, et al. Do the daily experiences of healthy men and women vary according to occupational prestige and work strain? *Psychosom Med* 2000;62:346–53. doi:10.1097/00006842-200005000-00008

For peer review only



For peer review only - http://bmjopen.bmj.com/site/about/guidelines.xhtml

32. De Raeve L, Jansen NWH, van den Brandt PA, et al. Interpersonal conflicts at work as a predictor of self-reported health outcomes and occupational mobility. *Occup Environ Med* 2007;66:16–22. doi:10.1136/oem.2007.037655

33. Haukenes I, Löve J, Hensing G, et al. Inequality in disability: an intersectional analysis of the co-constitution of gender, education and age. The Hordaland Health Study. *Crit Public Health* 2018;29(3):302–13. doi:10.1080/09581596.2018.1469730

34. Bauer GR. Incorporating intersectionality theory into population health research methodology: challenges and the potential to advance health equity. *Soc Sci Med* 2014;110:10–17. doi:10.1016/j.socscimed.2014.03.022

35. Berntsson L, Lundberg U, Krantz G. Gender differences in work-home interplay and symptom perception among Swedish white-collar employees. *J Epidemiol Community Health* 2006;60:1070–6. doi:10.1136/jech.2005.042192

36. Lidwall U, Marklund S. What is health work for women and men? A case-control study of gender-and sector-specific effects of psycho-social working conditions on long-term sickness absence. *Work* 2006;27(2):153–63.

37. Laasksonen M, Martikainen P, Rahkonen O, et al. Explanations for gender differences in sickness absence: evidence from middle-aged municipal employees from Finland. *Occup Environ Med* 2008;65:325–30. doi:10.1136/oem.2007.033910

38. Hensing G, Alexanderson K, Allebeck P, et al. Sick leave due to psychiatric disorder: higher incidence among women and longer duration for men. *Br J Psychiatry* 1996;169:740–6. doi:10.1192/bjp.169.6.740

39. Sandanger I, Nygård JF, Brage S, et al. Relation between health problems and sickness absence: Gender and age differences. A comparison of low-back pain,

For peer review only



For peer review only - http://bmjopen.bmj.com/site/about/guidelines.xhtml

psychiatric disorders and injuries. *Scand J Public Health* 2000;28:244–52. doi:10.1177/14034948000280040601

40. Schwartz SH, Lifschitz TR. Sex differences in value priorities: cross-cultural and multimethod studies. Journal of Personality ad Social Psychology. 2006;89(6):1010-28. Doi:  10.1037/0022-3514.89.6.1010

41. Courtenay WH. Constructions of masculinity and their influence on men's well-being: a theory of gender and health. *Soc Sci Med* 2000;50(10):1385–401. doi:10.1016/S0277-9536(99)00390-1

42. Peterson M. What men and women value at work: implications for workplace health. *Gender Med* 2004;1(2):106–24. doi:10.1016/S1550-8579(04)80016-0

43. Thompson AE, Anisimowicz Y, Miedema B, et al. The influence of gender and other patient characteristics on health care-seeking behavior: a QUALICOPC study. *BMC Fam Pract* 2016;17:38. doi:10.1186/s12875-016-0440-0

44. Slany C, Schütte S, Chastang J-F, et al. Psychosocial work factors and long sickness absence in Europe. *Int J Occup Environ Health* 2014;20(1):16–25. doi:10.1179/2049396713Y.0000000048

For peer review only



For peer review only - http://bmjopen.bmj.com/site/about/guidelines.xhtml

For peer review only



For peer review only - http://bmjopen.bmj.com/site/about/guidelines.xhtml

For peer review only



For peer review only - http://bmjopen.bmj.com/site/about/guidelines.xhtml

Online supplementary file A1. Gender-stratified association between occupational prestige and number of sickness absence days at follow-up. Incidence rate ratios (IRR) and 95% confidence intervals (95%CI) obtained from generalized estimating equation (GEE) with negative binomial regression

| | | Crude<br>IRR (95%CI) | Model I<br>IRR (95%CI) | Model II<br>IRR (95%CI) | Model III<br>IRR (95%CI) | Model IV<br>IRR (95%CI) |
|---|---|---|---|---|---|---|
| Female | N = 48029<br>n (weighted %) | | | | | |
| Occupational prestige | | | | | | |
| Low | 17150 (34.8) | 2.03 (2.00-2.06) | 1.85 (1.82-1.88) | 1.48 (1.45-1.51) | 1.24 (1.19-1.28) | 1.22 (1.18-1.27) |
| Medium | 15284 (32.1) | 1.20 (1.18-1.22) | 1.12 (1.11-1.14) | 1.01 (0.99-1.03) | 0.99 (0.97-1.01) | 1.05 (1.02-1.07) |
| High | 15595 (33.1) | 1.00 | 1.00 | 1.00 | 1.00 | 1.00 |
| Male | N = 49368<br>n (weighted %) | | | | | |
| Occupational prestige | | | | | | |
| Low | 15193 (30.4) | 3.56 (3.47-3.64) | 3.08 (3.01-3.16) | 1.55 (1.50-1.60) | 0.97 (0.94-1.01) | 1.00 (0.96-1.05) |

For peer review only

| | | | | | | |
|---|---|---|---|---|---|---|
| Medium | 19272 (38.9) | 2.13 (2.08-2.18) | 2.05 (2.00-2.11) | 1.21 (1.17-1.25) | 0.88 (0.85-0.91) | 0.92 (0.89-0.95) |
| High | 14903 (30.7) | 1.00 | 1.00 | 1.00 | 1.00 | 1.00 |

Model I adjusted for survey year, age, marital status, and previous sickness; Model II, additionally adjusting for education and income; Model III additionally adjusting for occupational class; Model IV additionally adjusting for employment type, contract type, and employment sector

For peer review only

Online supplementary file A2. Intersections of gender and occupational prestige and the association with long-term sickness absence among those with at least one spell of sickness absence. Odds ratios (OR) and 95% confidence intervals (95%CI) obtained from generalized estimating equation (GEE) with logistic regression

| | Total N = 21836 | Crude | Model I | Model II | Model III | Model IV |
|---|---|---|---|---|---|---|
| | n (weighted %) | OR (95%CI) | OR (95%CI) | OR (95% CI) | OR (95% CI) | OR (95%CI) |
| Female/low occupational prestige | 6098 (26.7) | 2.03 (1.96-2.11) | 1.70 (1.64-1.77) | 1.05 (1.00-1.09) | 1.11 (1.06-1.16) | 1.05 (1.00-1.11) |
| Female/medium occupational prestige | 4020 (18.1) | 1.48 (1.43-1.54) | 1.30 (1.25-1.35) | 0.93 (0.89-0.96) | 1.02 (0.98-1.06) | 1.02 (0.98-1.06) |
| Female/high occupational prestige | 3601 (16.3) | 1.44 (1.38-1.49) | 1.32 (1.27-1.30) | 1.08 (1.04-1.12) | 1.09 (1.05-1.14) | 1.06 (1.02-1.11) |
| Male/low occupational prestige | 3466 (16.7) | 1.89 (1.82-1.96) | 1.67 (1.61-1.70) | 1.22 (1.17-1.27) | 1.30 (1.24-1.37) | 1.31 (1.25-1.38) |
| Male/medium occupational prestige | 3236 (15.3) | 1.41 (1.36-1.47) | 1.30 (1.25-1.35) | 1.08 (1.03-1.12) | 1.16 (1.11-1.21) | 1.19 (1.13-1.24) |
| Male/high occupational prestige | 1415 (6.8) | 1.00 | 1.00 | 1.00 | 1.00 | 1.00 |

Model I adjusted for survey year, age, marital status, and previous sickness; Model II, additionally adjusting for education and income; Model III additionally adjusting for occupational class; Model IV additionally adjusting for employment type, contract type, and employment sector

For peer review only

**STROBE 2007 (v4) Statement—Checklist of items that should be included in reports of *cohort studies***

| Section/Topic | Item # | Recommendation | Reported on page # |
|---|---|---|---|
| **Title and abstract** | 1 | (*a*) Indicate the study's design with a commonly used term in the title or the abstract | 1 |
| | | (*b*) Provide in the abstract an informative and balanced summary of what was done and what was found | 2-3 |
| **Introduction** | | | |
| Background/rationale | 2 | Explain the scientific background and rationale for the investigation being reported | 4-6 |
| Objectives | 3 | State specific objectives, including any prespecified hypotheses | 6 |
| **Methods** | | | |
| Study design | 4 | Present key elements of study design early in the paper | 7 |
| Setting | 5 | Describe the setting, locations, and relevant dates, including periods of recruitment, exposure, follow-up, and data collection | 6-7 |
| Participants | 6 | (*a*) Give the eligibility criteria, and the sources and methods of selection of participants. Describe methods of follow-up | 7 |
| | | (*b*) For matched studies, give matching criteria and number of exposed and unexposed | |
| Variables | 7 | Clearly define all outcomes, exposures, predictors, potential confounders, and effect modifiers. Give diagnostic criteria, if applicable | 8-9 |
| Data sources/ measurement | 8* | For each variable of interest, give sources of data and details of methods of assessment (measurement). Describe comparability of assessment methods if there is more than one group | 8-9 |
| Bias | 9 | Describe any efforts to address potential sources of bias | 10 |
| Study size | 10 | Explain how the study size was arrived at | 7 |
| Quantitative variables | 11 | Explain how quantitative variables were handled in the analyses. If applicable, describe which groupings were chosen and why | 9 |
| Statistical methods | 12 | (*a*) Describe all statistical methods, including those used to control for confounding | 9-10 |
| | | (*b*) Describe any methods used to examine subgroups and interactions | |
| | | (*c*) Explain how missing data were addressed | 7 |
| | | (*d*) If applicable, explain how loss to follow-up was addressed | 7 |
| | | (*e*) Describe any sensitivity analyses | 10 |
| **Results** | | | |

For peer review only

| | | | |
|---|---|---|---|
| Participants | 13* | (a) Report numbers of individuals at each stage of study—eg numbers potentially eligible, examined for eligibility, confirmed eligible, included in the study, completing follow-up, and analysed | |
| | | (b) Give reasons for non-participation at each stage | |
| | | (c) Consider use of a flow diagram | |
| Descriptive data | 14* | (a) Give characteristics of study participants (eg demographic, clinical, social) and information on exposures and potential confounders | 15 |
| | | (b) Indicate number of participants with missing data for each variable of interest | |
| | | (c) Summarise follow-up time (eg, average and total amount) | 15 |
| Outcome data | 15* | Report numbers of outcome events or summary measures over time | 15 |
| Main results | 16 | (*a*) Give unadjusted estimates and, if applicable, confounder-adjusted estimates and their precision (eg, 95% confidence interval). Make clear which confounders were adjusted for and why they were included | 15-16 |
| | | (*b*) Report category boundaries when continuous variables were categorized | |
| | | (*c*) If relevant, consider translating estimates of relative risk into absolute risk for a meaningful time period | |
| Other analyses | 17 | Report other analyses done—eg analyses of subgroups and interactions, and sensitivity analyses | 19 |
| **Discussion** | | | |
| Key results | 18 | Summarise key results with reference to study objectives | 23 |
| **Limitations** | | | |
| Interpretation | 20 | Give a cautious overall interpretation of results considering objectives, limitations, multiplicity of analyses, results from similar studies, and other relevant evidence | 23-25 |
| Generalisability | 21 | Discuss the generalisability (external validity) of the study results | 25 |
| **Other information** | | | |
| Funding | 22 | Give the source of funding and the role of the funders for the present study and, if applicable, for the original study on which the present article is based | 27 |

*Give information separately for cases and controls in case-control studies and, if applicable, for exposed and unexposed groups in cohort and cross-sectional studies.

**Note:** An Explanation and Elaboration article discusses each checklist item and gives methodological background and published examples of transparent reporting. The STROBE checklist is best used in conjunction with this article (freely available on the Web sites of PLoS Medicine at http://www.plosmedicine.org/, Annals of Internal Medicine at http://www.annals.org/, and Epidemiology at http://www.epidem.com/). Information on the STROBE Initiative is available at www.strobe-statement.org.

For peer review only

# Occupational prestige and sickness absence inequality in employed women and men in Sweden: a registry-based study

| | |
|---|---|
| Journal: | *BMJ Open* |
| Manuscript ID | bmjopen-2021-050191.R1 |
| Article Type: | Original research |
| Date Submitted by the Author: | 15-May-2021 |
| Complete List of Authors: | Nwaru, Chioma; University of Gothenburg, School of Public Health and Community Medicine, Institute of Medicine, Sahlgrenska Academy<br>Berglund, Tomas; University of Gothenburg, Department of Sociology and Work Science<br>Hensing, Gunnel; University of Gothenburg, School of Public Health and Community Medicine, Institute of Medicine, Sahlgrenska Academy |
| <b>Primary Subject Heading</b>: | Public health |
| Secondary Subject Heading: | Epidemiology, Mental health, Occupational and environmental medicine |
| Keywords: | SOCIAL MEDICINE, EPIDEMIOLOGY, OCCUPATIONAL & INDUSTRIAL MEDICINE |
| | |

SCHOLARONE™ Manuscripts

*I, the Submitting Author has the right to grant and does grant on behalf of all authors of the Work (as defined in the below author licence), an exclusive licence and/or a non-exclusive licence for contributions from authors who are: i) UK Crown employees; ii) where BMJ has agreed a CC-BY licence shall apply, and/or iii) in accordance with the terms applicable for US Federal Government officers or employees acting as part of their official duties; on a worldwide, perpetual, irrevocable, royalty-free basis to BMJ Publishing Group Ltd ("BMJ") its licensees and where the relevant Journal is co-owned by BMJ to the co-owners of the Journal, to publish the Work in this journal and any other BMJ products and to exploit all rights, as set out in our licence.*

*The Submitting Author accepts and understands that any supply made under these terms is made by BMJ to the Submitting Author unless you are acting as an employee on behalf of your employer or a postgraduate student of an affiliated institution which is paying any applicable article publishing charge ("APC") for Open Access articles. Where the Submitting Author wishes to make the Work available on an Open Access basis (and intends to pay the relevant APC), the terms of reuse of such Open Access shall be governed by a Creative Commons licence – details of these licences and which Creative Commons licence will apply to this Work are set out in our licence referred to above.*

*Other than as permitted in any relevant BMJ Author's Self Archiving Policies, I confirm this Work has not been accepted for publication elsewhere, is not being considered for publication elsewhere and does not duplicate material already published. I confirm all authors consent to publication of this Work and authorise the granting of this licence.*

For peer review only

# Occupational prestige and sickness absence inequality in employed women and men in Sweden: a registry-based study

Chioma A. Nwaru,[1] Tomas Berglund,[2] Gunnel Hensing[1]

[1]School of Public Health and Community Medicine, Institute of Medicine, Sahlgrenska Academy, University of Gothenburg, Gothenburg, Sweden

[2]Department of Sociology and Work Science, University of Gothenburg, Gothenburg, Sweden.

**Correspondence to** Chioma A. Nwaru, School of Public Health and Community Medicine, Institute of Medicine, Sahlgrenska Academy, University of Gothenburg, Box 414, 40530, Gothenburg, Sweden; chioma.nwaru@gu.se

**Key words:** occupational prestige, gender, sickness absence

**Word count**
Abstract: 300
Main text: 4172



For peer review only - http://bmjopen.bmj.com/site/about/guidelines.xhtml

# ABSTRACT

**Objectives** Socioeconomic position has been linked to sickness absence. However, less is known about the role of occupational prestige, a measure of social status afforded by one's occupation, in sickness absence. We investigated the association between occupational prestige and sickness absence and the distribution of the association in women and men. We also examined the effect of intersections of gender and occupational prestige on sickness absence.

**Design** Longitudinal

**Setting** A nationwide representative sample of Swedish working population.

**Participants** 97 397 employed individuals aged 25-59 years selected from the 2004, 2007 and 2010 waves of the Swedish Labour Force Survey and prospectively linked to the Swedish Longitudinal Integration Database for Health Insurance and Labour Market Studies.

**Outcome measures** The number of sickness absence days in any particular year during a 3-year follow-up and long-term (>120 days) sickness absence based on those with at least one sick leave spell during the follow-up.

**Results** Occupational prestige was weakly associated with sickness absence in the total sample after adjusting for potential confounders. In the gender-stratified analysis, women in lower prestige occupations had higher absenteeism rates than women in high prestige occupations; men in lower prestige occupations had higher odds for long-term SA than men in high prestige occupations. In the intersectional analysis, women regardless of prestige level and men in lower prestige occupations had higher probability of sickness absence compared with men in high prestige occupations. Women in high prestige occupations had the highest absenteeism rates (IRR, 2.25, 95%CI, 2.20-2.31), while men in medium prestige occupations had the lowest rates (IRR, 1.17, 95%CI, 1.13-1.20). Compared with the rest of the groups, men in low and medium prestige occupations had higher odds for long-term absence.




For peer review only - http://bmjopen.bmj.com/site/about/guidelines.xhtml

**Conclusions** There is need to pay close attention to occupational prestige as a factor that may influence health and labour market participation.

For peer review only



For peer review only - http://bmjopen.bmj.com/site/about/guidelines.xhtml

## Strengths and limitations of this study

- A large, reliable, nationally representative sample of employed women and men in Sweden was used for the study.
- Data on sickness absence was register-based.
- The incorporation of gender-occupational prestige intersections expanded previous knowledge on gender differences in sickness absence.
- Occupational prestige was based on job titles provided in the inclusion year, which may not necessarily be the longest held occupations for some of the participants.
- The study did not include sickness absence lasting 14 days or less.

For peer review only



For peer review only - http://bmjopen.bmj.com/site/about/guidelines.xhtml

# INTRODUCTION

Sickness absence (SA), particularly long-term absence, has consistently been associated with unemployment,[1] disability retirement,[2] and mortality,[3] and could lead to permanent exit from the workforce. Socioeconomic position (SEP) is an important determinant of SA. Occupation, income, and education are key indicators of SEP. Compared with education and income, occupation is a more complex factor; it does not have a single measure that captures the occupation-health relationship.[4] Previous studies on occupation and SA have used occupational classifications based on employment relations[5] and job titles.[6] Other studies have combined job titles, education, and similarity of work content.[7] Several occupational classification schemes exist that have not been studied in relation to SA. Occupation-based measures originate from different theoretical concepts, and each classification tends to capture a specific aspect of occupation related to health. Moreover, the mechanisms connecting occupation to health are not similar across different occupation-based measurements.[4, 8] To better understand the role of various aspects of occupation on SA, which is important for creating interventions to reduce SA, an exploration of different occupation-based measures, particularly those that have received less attention, is warranted.

The present study focuses on the role of occupational prestige as a determinant of SA. Occupational prestige as used here reflects social status, given that the measure represents the value placed on different occupations by the society. Occupational prestige is measured using the Standard International Occupational Prestige Scale (SIPOS), which is a scale that provides hierarchical ranking from least to most esteemed occupations according to average societal ratings.[4, 9] No studies so far have investigated the association between occupation and SA using SIOPS-based occupational prestige as an indicator. Investigating this association can help to capture features of social hierarchy (acceptance, respect, recognition, admiration, autonomy, power, social network and social support)[10, 11] that may not be adequately



For peer review only - http://bmjopen.bmj.com/site/about/guidelines.xhtml

represented in other measures of occupational classification, such as occupational class, thus illuminating how social position and its associated rewards and privileges contribute in shaping health risk.

The position a given occupation has on the prestige scale strongly impacts both the adoption of an occupational identity and construction of self-esteem.[12-14] Compared to individuals in lower prestige occupations, those in high prestige occupations are more likely to identify with their occupations because of the social rewards and privileges associated with high status occupations.[12, 13] Owing to a lack of such rewards and privileges, employees in lower prestige occupations may have increased risk of work alienation (i.e., psychological detachment from their jobs), low self-esteem, poor work satisfaction, and reduced social interactions.[13, 14] These negative affects can induce poor response to stress and consequently increase the risk of poor health.[15, 16] Previous research has shown that occupational prestige is a determinant of different health outcomes, and that it can influence health independently of other SEP indicators and job characteristics. For example, Fujishuro *et al.*[17] reported increased poor self-rated health among employees in lower prestige occupations after controlling for occupational categories, education, income, job stress, workplace social support, and job satisfaction. Additionally, occupational prestige was found to be a determinant of self-rated health[18] as well as a determinant of mortality,[19] even when controlling for education and income in both studies. In line with these studies, we expect to find increased risk of SA among employees working in lower prestige occupations.

Gender is an important determinant of SA. A common statistical approach when investigating gender differences in SA is to compare the risk in women and men, while controlling for potential confounders. Thus, in several studies women (and men) are treated as a homogenous group.[20,21] Hankivsky et al.[22] noted that such an approach fails to recognize the diversity of women (and men) regarding other forms of social identities and the



For peer review only - http://bmjopen.bmj.com/site/about/guidelines.xhtml

relational nature among different social identities. They suggested that researchers incorporate intersectional approach, i.e., investigate the extent to which gender combines with other social identities to create health inequality. The intersectional approach integrates both between and within group differences,[23] therefore offering the potential to provide insight on different levels of SA inequality.

Our aims in this study are to (1) investigate whether occupational prestige is associated with SA, (2) examine the distribution of this association among women and men, and (3) investigate the effects of intersections of gender and occupational prestige on SA. The current study has the potential to improve knowledge on determinants of SA as well as on target groups, both of which are crucial for reducing SA and improving labour market participation.

# METHODS

## Study population and design

This study is part of the New Ways and the Polarization programs at the University of Gothenburg. The study is based on data of participants in the Swedish Labour Force Survey (LFS), which is a quarterly survey used to monitor labour market developments for the entire population aged 15–74 years. The LFS utilizes a rotating sampling design, meaning that participants in the survey are interviewed once every quarter for a maximum of eight times over a 2-year period, after which they are replaced with new sample individuals.[24] For this study, we selected from three cross-sections at the end of the survey years 2004, 2007, and 2010, individuals aged 25–59 years who were employed wage earners according to their main economic activity and were at work, i.e., not self-employed, unemployed, retired or on disability pension (N = 107 608). Using the participants' encrypted identification number, we



For peer review only - http://bmjopen.bmj.com/site/about/guidelines.xhtml

linked information from the cohorts with the LISA (Swedish Longitudinal Integration Database for Health Insurance and Labour Market Studies) register[25] and followed each cohort for 3 years thus covering the period 2005–2013. We excluded from the cohorts those participants who started receiving old age or disability pension by the end of the study period (n = 6278). We also excluded participants with incomplete follow-up data and those with missing exposure or potential control variables (n = 3933), resulting in a final working sample of 97 397 participants (Figure 1).

Patient and public involvement

This study was performed without patient or public involvement. The Swedish national registers are protected by special legislation that makes it possible for researchers to collect certain information without personal consent.

Ethical approval

The regional ethical review board in Gothenburg approved the study (Dnr: 090-17). All the data used in this study were anonymised and the researchers do not have access to any personal information that could identify individuals included in the study.

## Measurements

Occupational prestige

We measured occupational prestige, the exposure in this study, with the SIOPS.[4,9] The scores, which ranged from 13 to 78, were calculated based on occupational prestige studies conducted in more than 60 countries. Respondents in each of the participating countries were asked to rate a set of occupational titles with respect to their social position. The ratings were aggregated into mean scores and reported as indicators of the relative prestige scores of the evaluated occupations.[26] Svensson and Ulfsdotter[27] compared the SIOPS and occupational prestige scores generated from a Swedish population and found a



For peer review only - http://bmjopen.bmj.com/site/about/guidelines.xhtml

high correlation (0.8) between the scores. For this study, we produced the prestige scores by assigning an occupational prestige score (i.e., the SIOPS) to each occupational title provided by the respondents (obtained from the LISA) during the inclusion (i.e., 2004, 2007, and 2010) based on a four-level ISCO-88 (International Standard Classification of Occupations, revision 1988) classification. Based on the distribution of occupational prestige in the current data, we divided the scores into tertiles (15–34 points, 35–50 points, and 51–78 points) to represent low, medium and high occupational prestige categories, respectively. The last category was used as the reference in the analyses. We constructed six intersectional groups using information on the prestige groups and on gender (obtained from the LFS). The groups were women in low prestige occupations, women in medium prestige occupations, women in high prestige occupations, men in low prestige occupations, men in medium prestige occupations, and men in high prestige occupations (reference group). Table 1 provides the five most common high prestige scores and examples of occupations in each prestige category for women and men.

Sickness absence

In Sweden, all individuals aged 16 years and above with an income from work or unemployment benefits are entitled to sickness absence benefits. If the sick leave lasts for more than seven consecutive days, starting from the eighth day, a doctor's certificate is required. During the study observation years (2005–2013), the employers paid for absence days for 14 days or less after a qualifying day without benefits. From day 15 onward, the Social Insurance Agency (SIA) pays SA benefits. Unemployed individuals have one qualifying day and receive sick pay from the SIA from the second day. In this study, information on SA was based on data from the LISA that only contains information on SA from day 15 onward. We used two measures of SA: the number of SA days in any particular year during the follow-up and long-term SA. The latter was based on those with at least one



For peer review only - http://bmjopen.bmj.com/site/about/guidelines.xhtml

sick leave spell during the follow-up and defined as absence days lasting over 120 days. In the so-called rehabilitation chain in Sweden, evaluation of work capacity and the right to SA benefit are assessed at specific times, starting from day 90 into the SA period. By choosing 120 days as the cut point when the individuals would have undertaken the first assessment, our measure of long-term SA is more likely to represent serious health conditions, which are of interest in this study.

Potential confounders

From the LFS database, we extracted information on participants' age (25–34, 35–44, and 45–59 years), marital status (single, cohabiting, and married), occupational class (manual, lower non-manual, and higher non-manual), employment sector (private or public), employment type (temporary or permanent), and contract type (full-time, part-time) at the time of inclusion in the study. From the LISA register, we obtained information on previous SA for the 2 years preceding the study observation period. We also retrieved information on highest education attained (primary, secondary, or tertiary) and on gross annual salaries (divided into quartiles) of the participants during the inclusion year from the LISA database. We categorized the year of measurement as 2004, 2007, and 2010. We treated these variables as confounders capable of influencing the association between occupational prestige and SA.

For peer review only

Table 1: List of five most common high prestige scores and examples of occupations in each prestige category for women and men

| Five most common high prestige scores (SIOPS) among women | Examples of common occupations in each category according to International Standard Classification of Occupations, revision 1988 (ISCO-88) |
|---|---|
| 52 | Social work professions, life science technicians, managers of small enterprises in health and social work |
| 53 | Administrative secretaries and related associate professionals, computer assistants |
| 54 | District nurses, midwives, head nurses, Liberians and related information professionals |
| 57 | Primary education teaching professionals, market research analysts and related professionals |
| 60 | Teaching professionals, artistic and practical subjects, teaching professionals, academic subjects, production and operations managers in education |
| **Five most common high prestige scores (SIOPS) among men** | |
| 51 | Computer systems designers, analysts and programmers, computing professionals not elsewhere classified |
| 53 | Computer assistants, administrative secretaries and related associate professionals |
| 57 | Primary education teaching professionals, business professionals not elsewhere classified, market research analysts and related professionals |



For peer review only - http://bmjopen.bmj.com/site/about/guidelines.xhtml

| | |
|---|---|
| 60 | Teaching professionals, artistic and practical subjects, teaching professionals, academic subjects, finance and administrative managers, sales and marketing managers, vocational teaching professionals |
| 78 | Medical doctors, college, university and higher education teaching professionals |

For peer review only



For peer review only - http://bmjopen.bmj.com/site/about/guidelines.xhtml

## Statistical analyses

Data analyses were performed with the generalized estimating equation (GEE), a statistical technique suitable for modelling repeated outcome variables. The technique takes into account the within-subject correlation of the repeated SA variable.[28] The annual rate of SA days was estimated using GEE with negative binomial. The negative binomial distribution was chosen because of the overdispersion in the count outcome variable.[29] Exchangeable correlation was specified in all the models as the correlation structure based on the result of a preliminary investigation. However, GEE is robust to misspecification of the correlation structure.[28] Six separate models were estimated, starting with an age-adjusted model followed by five other adjusted models. In Model I, survey year, gender, and marital status were entered into the model. In Model II, previous SA was additionally included into the model. In order to estimate the effect of occupational prestige on SA in the presence of other commonly used SEP indicators, additional adjustment was made for income and education in Model III, and occupational class in Model IV. Employment-related characteristics, which we interpreted as proxy measures for work-related characteristics, were added in the final model (Model V). The coefficients were expressed as incidence rate ratios (IRRs) and 95% confidence intervals (CIs).

GEE with logistic regression was used to estimate the annual risk of having >120 SA days during the three-year follow-up. The modelling strategy was the same as for the count outcome. The robustness of the results regarding long-term SA was tested by repeating the analyses using 90 days as the cut point for long-term SA since the Swedish evaluation of work capacity within the rehabilitation framework starts at day 90. All the analyses were population weighted to correct for biases due to sampling. IBM SPSS version 26 for Windows was used for all the statistical analyses.



For peer review only - http://bmjopen.bmj.com/site/about/guidelines.xhtml

Table 2. Distribution of sample characteristics by occupational prestige at baseline and the association of the characteristics with sickness absence at follow-up

| | Total | Occupational prestige at baseline | | | | Incidence rate ratios (IRR) associated with number of SA days at follow-up | Odds ratios (OR) associated with long-term sickness absence at follow-up |
|---|---|---|---|---|---|---|---|
| | N = 97397 | | | | | N = 97397 | N = 21836† |
| | | Low (n = 32343) | Medium (n = 34556) | High (n = 30498) | p-value | | |
| | n (weighted %) | Weighted % | Weighted % | Weighted % | | IRR (95%CI) | OR (95%CI) |
| Survey year | | | | | 0.000 | | |
| 2004 | 27950 (34.1) | 32.2 | 36.7 | 31.0 | | 1.32 (1.31-1.34) | 1.48 (1.45-1.50) |



For peer review only - http://bmjopen.bmj.com/site/about/guidelines.xhtml

| | | | | | | | |
|---|---|---|---|---|---|---|---|
| 2007 | 31483 (32.8) | 32.8 | 35.3 | 31.9 | | 0.88 (0.87-0.89) | 0.97 (0.96-0.99) |
| 2010 | 37964 (33.1) | 32.5 | 34.9 | 32.6 | | 1.00 | 1.00 |
| Age (years) | | | | | 0.000 | | |
| 25-34 | 26259 (27.7) | 34.2 | 35.3 | 30.5 | | 1.00 | 1.00 |
| 35-44 | 30890 (32.1) | 31.0 | 36.2 | 32.8 | | 1.32 (1.30-1.34) | 1.38 (1.35-1.41) |
| 45-59 | 40248 (40.2) | 32.5 | 35.5 | 32.0 | | 1.63 (1.61-1.65) | 1.49 (1.46-1.51) |
| Gender | | | | | 0.000 | | |
| Women | 48029 (47.5) | 34.8 | 32.1 | 33.1 | | 1.87 (1.85-1.89) | 1.11 (1.09-1.13) |
| Men | 49368 (52.5) | 30.4 | 38.9 | 30.7 | | 1.00 | 1.00 |
| Marital status | | | | | 0.000 | | |
| Single | 23441 (24.3) | 37.2 | 34.8 | 28.0 | | 1.25 (1.24-1.26) | 1.29 (1.27-1.31) |
| Cohabiting | 28386 (28.9) | 33.7 | 37.6 | 28.7 | | 0.93 (0.92-0.94) | 0.95 (0.93-0.96) |
| Married | 45570 (46.8) | 29.3 | 34.9 | 35.8 | | 1.00 | 1.00 |
| Education | | | | | 0.000 | | |
| Primary | 9668 (10.0) | 59.5 | 33.5 | 7.0 | | 2.16 (2.13-2.20) | 1.56 (1.53-1.59) |
| Secondary | 46415 (47.2) | 47.2 | 39.7 | 13.1 | | 1.57 (1.55-1.59) | 1.21 (1.19-1.23) |
| Tertiary | 41314 (42.7) | 10.0 | 31.7 | 58.4 | | 1.00 | 1.00 |



For peer review only - http://bmjopen.bmj.com/site/about/guidelines.xhtml

| | | | | | | | |
|---|---|---|---|---|---|---|---|
| Occupational class | | | | | 0.000 | | |
| Manual | 40389 (40.8) | 73.7 | 26.2 | 0.2 | | 1.00 | 1.00 |
| Lower non-manual | 38522 (39.5) | 6.0 | 61.2 | 32.8 | | 0.58 (0.57-0.58) | 0.75 (0.73-0.76) |
| Higher non-manual | 18486 (19.7) | 0.1 | 4.2 | 95.7 | | 0.38 (0.37-0.38) | 0.70 (0.69-0.72) |
| Individual income | | | | | 0.000 | | |
| Lowest | 24365 (25.2) | 54.3 | 29.5 | 16.2 | | 5.63 (5.54-5.71) | 3.40 (3.31-3.49) |
| Second quartile | 24369 (24.8) | 41.0 | 38.7 | 20.3 | | 3.12 (3.07-3.17) | 1.92 (1.87-1.97) |
| Third quartile | 24330 (24.5) | 26.1 | 42.1 | 31.9 | | 2.18 (2.15-2.22) | 1.58 (1.53-1.62) |
| Highest | 24333 (25.5) | 8.9 | 32.6 | 58.5 | | 1.00 | 1.00 |
| Employment sector | | | | | 0.000 | | |
| Private | 63136 (66.5) | 31.9 | 41.5 | 26.6 | | 1.00 | 1.00 |
| Public | 34261 (33.5) | 33.7 | 24.0 | 42.3 | | 1.65 (1.63-1.67) | 1.27 (1.25-1.29) |
| Employment type | | | | | 0.000 | | |
| Temporary | 8764 (9.1) | 41.5 | 25.9 | 32.6 | | 1.14 (1.12-1.16) | 1.17 (1.15-1.20) |
| Permanent | 88633 (90.9) | 31.6 | 36.6 | 31.8 | | 1.00 | 1.00 |
| Contract type | | | | | 0.000 | | |
| Part-time | 19641 (19.3) | 49.6 | 27.4 | 23.0 | | 1.60 (1.58-1.61) | 1.33 (1.31-1.35) |

For peer review only

| | | | | | | | |
|---|---|---|---|---|---|---|---|
| Full-time | 77756 (80.7) | 28.4 | 37.6 | 34.0 | | 1.00 | 1.00 |
| Previous SA in the last two years | | | | | 0.000 | | |
| No | 81995 (84.8) | 30.4 | 36.1 | 33.6 | | 1.00 | 1.00 |
| Yes | 15402 (15.2) | 44.4 | 33.4 | 22.3 | | 5.04 (4.99-5.09) | 2.45 (2.42-2.49) |

† Analyses performed among those with at least one spell of registered sickness absence during follow-up



For peer review only - http://bmjopen.bmj.com/site/about/guidelines.xhtml

# RESULTS

## Descriptive statistics

The mean age of the study population was 42 years (SD, 9.7 years). The participants' characteristics, overall and by occupational prestige groups, are presented in table 2. There were more women (than men) in low prestige occupations; the same applied for temporary (versus permanent) and part-time (versus full-time) employees, and those with previous SA (versus those without). Working in high prestige occupations was prevalent among those in higher non-manual occupations, those with a tertiary education, and those in the highest income quartiles. During the study follow-up, 22% (21 836) of the participants had at least one episode of SA lasting more than 14 days. Sixteen percent (n = 3709) of the 21 836 had at least one episode of SA lasting over 120 days. All participants' characteristics were associated with both the number of SA days and long-term absence at follow-up (table 2).

## Occupational prestige differences in number of sickness absence days

Table 3 shows the IRRs for the association between occupational prestige and number of SA days in the total sample. There was a graded association between occupational prestige and SA in the age-adjusted model. Employees in low prestige occupations had over two times more SA than those in high prestige group. Those in medium prestige occupations had 1.41 times more SA than employees in high prestige group. The IRRs increased after adding survey year, gender, and marital status into the model. Additional adjustment for previous SA attenuated the IRRs for both groups. Additional adjustment for income and education increased the IRR for employees in low prestige occupations and significantly attenuated the IRR for employees in medium prestige occupations. Additional adjustment for occupational



For peer review only - http://bmjopen.bmj.com/site/about/guidelines.xhtml

class eliminated the increased IRR among employees in lower prestige occupations. Adjustment for employment-related variables did not seem to have much influence on the association after adjustment for sociodemographic and socioeconomic factors.

When the data were analysed separately for women and men, the result from the age-adjusted model (online supplementary table A1) showed significant occupational prestige differences in both genders. As with the total sample, additional adjustment for income and education (Model III) and occupational class (Model IV) significantly reduced the estimates. After adjusting for all potential confounders (Model V), among women, the IRR remained statistically significant among women in low (IRR, 1.22; 95% CI, 1.18–1.27) and medium (IRR, 1.05; 95% CI, 1.02–1.07) occupational prestige groups compared with women in the high occupational prestige group. Among men, no increased IRR was found both for men in low (IRR, 1.00; 95% CI, 0.96–1.05) and men in medium (IRR, 0.92; 95% CI, 0.89–0.95) prestige occupations compared with men in high prestige occupations.

Table 4 presents the distribution (weighted %) of intersectional groups and the IRR across the groups, with men in the high occupational prestige group as the reference group. All the groups had increased absence rates compared with men in high prestige occupations in the age-adjusted model. After accounting for all potential confounders, women in high prestige occupations had the highest absence rates (IRR, 2.25, 95% CI, 2.20–2.31), which is an unexpected finding in this study. Women in medium prestige occupations had the second highest absence rate (IRR, 2.01, 95% CI, 01.95–2.06) followed by women in low prestige occupations (IRR, 1.68, 95% CI, 1.62-1.74), men in low prestige occupations (IRR, 1.37, 95% CI, 1.32-1.42), and men in medium prestige occupations (IRR, 1.17, 95% CI, 1.13-1.20).



For peer review only - http://bmjopen.bmj.com/site/about/guidelines.xhtml

Table 3. Association of occupational prestige with number of sickness absence days at follow-up. Incidence rate ratios (IRR) and 95% confidence intervals (95%CI) obtained from generalized estimating equation (GEE) with negative binomial regression

| | Total N = 97397 | Age-adjusted | Model I | Model II | Model III | Model IV | Model V |
|---|---|---|---|---|---|---|---|
| | weighted % | IRR (95%CI) | IRR (95%CI) | IRR (95%CI) | IRR (95%CI) | IRR (95% CI) | IRR (95% CI) |
| Occupational prestige | | | | | | | |
| Low | 32.5 | 2.48 (2.45-2.51) | 2.68 (2.64-2.71) | 2.35 (2.32-2.39) | 1.44 (1.41-1.47) | 1.01 (0.98-1.04) | 1.02 (1.00-1.05) |
| Medium | 35.7 | 1.41 (1.40-1.43) | 1.61 (1.59-1.64) | 1.53 (1.50-1.55) | 1.12 (1.11-1.14) | 0.96 (0.94-0.98) | 1.00 (0.98-1.02) |
| High | 31.8 | 1.00 | 1.00 | 1.00 | 1.00 | 1.00 | 1.00 |

Model I, additionally adjusting for survey year, gender, and marital status; Model II, additionally adjusting for previous sickness; Model III, additionally adjusting for education and income; Model IV, additionally adjusting for occupational class; Model V, additionally adjusting for employment type, contract type, and employment sector



For peer review only - http://bmjopen.bmj.com/site/about/guidelines.xhtml

Table 4. Intersections of gender and occupational prestige and the association with number of sickness absence days at follow-up. Incidence rate ratios (IRR) and 95% confidence intervals (95%CI) obtained from generalized estimating equation (GEE) with negative binomial regression

| | Total N = 97397 | Age-adjusted | Model I | Model II | Model III | Model IV | Model V |
|---|---|---|---|---|---|---|---|
| | weighted % | IRR (95%CI) | IRR (95%CI) | IRR (95%CI) | IRR (95%CI) | IRR (95%CI) | IRR (95%CI) |
| Women/low prestige occup. | 16.5 | 6.06 (5.92-6.21) | 6.18 (6.04-6.32) | 4.91 (4.80-5.02) | 2.56 (2.49-2.64) | 1.76 (1.70-1.82) | 1.68 (1.62-1.74) |
| Women/medium prestige occup. | 15.2 | 3.59 (3.50-3.68) | 3.58 (3.50-3.67) | 3.00 (2.93-3.08) | 2.11 (2.06-2.17) | 1.98 (1.93-2.03) | 2.01 (1.95-2.06) |
| Women/high prestige occup. | 15.7 | 2.93 (2.86-3.01) | 2.99 (2.92-3,06) | 2.68 (2.61-2.74) | 2.38 (2.32-2.43) | 2.35 (2.30-2.41) | 2.25 (2.20-2.31) |
| Men/low prestige occup. | 16.0 | 3.60 (3.51-3.69) | 3.57 (3.48-3.65) | 3.11 (3.04-3.19) | 1.93 (1.88-1.99) | 1.34 (1.29-1.38) | 1.37 (1.32-1.42) |
| Men/medium prestige occup. | 20.4 | 2.15 (2.10-2.20) | 2.15 (2.10-2.21) | 2.06 (2.01-2.11) | 1.43 (1.39-1.47) | 1.12 (1.09-1.15) | 1.17 (1.13-1.20) |
| Men/high prestige occup. | 16.1 | 1.00 | 1.00 | 1.00 | 1.00 | 1.00 | 1.00 |

Model I, additionally adjusting for survey year and marital status; Model II, additionally adjusting for previous sickness; Model III, additionally adjusting for education and income; Model IV, additionally adjusting for occupational class; Model V, additionally adjusting for employment type, contract type, and employment sector

For peer review only

## Occupational prestige differences in long-term sickness absence

In the total sample, employees in lower prestige occupations had higher OR for long-term SA than those in high prestige occupations in the age-adjusted model (online supplementary table A2). However, after adjusting for all potential confounders, only the OR for employees in low prestige occupations showed slightly increased OR for long-term SA (OR, 1.07, 95%CI, 1.04-1.11). In the gender-stratified results seen in table 5, among men, there was a strong graded association between occupational prestige and long-term SA in the age-adjusted model. Additional adjustment for survey year and marital status (Model I), previous sickness absence (Model II), as well as education and income (Model III) had an attenuating effect, while adjustment for occupational class (Model IV) increased the ORs. After accounting for all potential confounders, men in low and medium prestige occupations had 26% and 14% increased odds, respectively, compared with men in high prestige occupations (Model V). Among women, there were no occupational prestige differences and no increased ORs with respect to long-term SA after accounting for all potential confounders (Model V).

The results with the intersectional groups showed age-adjusted ORs ranging from 1.43 to 2.09, with the highest OR found among women in low prestige occupations (online supplementary table A3). However, in the full model (Model V), men in low prestige occupations had the highest risk (OR, 1.31, 95% CI, 1.25-1.38), followed by men in medium prestige occupations (OR, 1.19, 95% CI, 1.13-1.24), women in high prestige occupations (OR, 1.06, 95% CI, 1.02-1.11), and women in low prestige occupations (OR, 1.05, 95% CI, 01.00-1.11). No statistically significant OR was found among women in medium prestige



For peer review only - http://bmjopen.bmj.com/site/about/guidelines.xhtml

occupations (OR, 1.02, 95%CI, 0.98-1.06). The estimates pointed toward the same direction when we repeated the analyses using 90 days as the cut point (data not shown).

For peer review only

For peer review only

Table 5. Gender-stratified association between occupational prestige and long-term sickness absence among those with at least one spell of sickness absence.

Odds ratios (OR) and 95% confidence intervals (95%CI) obtained from generalized estimating equation (GEE) with logistic regression

| | | Age-adjusted OR (95%CI) | Model I OR (95%CI) | Model II OR (95%CI) | Model III OR (95%CI) | Model IV OR (95%CI) | Model V OR (95%CI) |
|---|---|---|---|---|---|---|---|
| Women | N = 13719 weighted % | | | | | | |
| Occupational prestige | | | | | | | |
| Low | 43.7 | 1.40 (1.37-1.43) | 1.39 (1.36-1.42) | 1.30 (1.27-1.33) | 1.06 (1.03-1.09) | 1.01 (0.96-1.05) | 0.98 (0.94-1.03) |
| Medium | 29.6 | 1.03 (1.00-1.05) | 1.01 (0.99-1.04) | 0.99 (0.96-1.01) | 0.90 (0.87-0.92) | 0.95 (0.93-0.98) | 0.98 (0.95-1.01) |
| High | 26.7 | 1.00 | 1.00 | 1.00 | 1.00 | 1.00 | 1.00 |
| Men | N = 8117 weighted % | | | | | | |
| Occupational prestige | | | | | | | |
| Low | 43.0 | 1.92 (1.85-1.99) | 1.86 (1.79-1.93) | 1.64 (1.58-1.71) | 1.07 (1.02-1.12) | 1.26 (1.19-1.34) | 1.26 (1.19-1.34) |
| Medium | 39.4 | 1.42 (1.37-1.48) | 1.40 (1.35-1.46) | 1.28 (1.23-1.33) | 0.97 (0.92-1.01) | 1.12 (1.06-1.19) | 1.14 (1.08-1.21) |
| High | 17.6 | 1.00 | 1.00 | 1.00 | 1.00 | 1.00 | 1.00 |



For peer review only - http://bmjopen.bmj.com/site/about/guidelines.xhtml

For peer review only

Model I, additionally adjusting for survey year and marital status; Model II, additionally adjusting for previous sickness; Model III, additionally adjusting for education and income; Model IV, additionally adjusting for occupational class; Model V, additionally adjusting for employment type, contract type, and employment sector

For peer review only



For peer review only - http://bmjopen.bmj.com/site/about/guidelines.xhtml

# DISCUSSION

We found a weak association between occupational prestige and SA in the total sample after controlling for age, survey year, gender, marital status, previous SA, education, income, occupational class and employment-related variables. However, when we assessed the distribution of the association separately in women and men, we found a graded association between occupational prestige and SA in both genders. Women in lower prestige occupations had higher rates of SA days than women in high prestige occupations. Men in lower prestige occupations had higher odds for long-term SA than men in high prestige occupations. When we pooled the sample and investigated the effect of intersections of gender and occupational prestige on SA, we found that men in high prestige occupations had the lowest IRR for SA days, as well as the lowest OR for long-term SA than the rest of the groups. Of all the groups, women in high prestige occupations had the highest IRR for SA days, while men in low prestige occupations had the highest odds for long-term SA.

Our finding of increased SA among women and men in lower occupational prestige corroborates previous studies that used different health outcomes.[17-19] Occupational class, income, and education significantly impacted on the effect estimates, but the association remained statistically significant, thus supporting the notion that occupational prestige taps into aspects of the occupation-health relationship that are not represented by education, income, and occupational class.[17] Work stress has been hypothesized as one mechanism connecting lower occupational prestige and poor health outcomes. Hoven *et al.*[30] reported that employees in lower prestige occupations had increased work stress, i.e., effort-reward imbalance, which in turn, increased their risk of depressive symptoms. Working in lower prestige occupations may also induce feelings of low self-esteem[13, 17] that may lead to increased risk of anxiety, poor social functioning, risky behaviour,[31] and poor recovery

For peer review only

from illness.[32] Mattews *et al.*[33] found that employees in lower prestige occupations more often experienced interpersonal conflicts, boredom, and increased heart rate; and interpersonal conflicts at work have been associated with increased risk of prolonged fatigue and poor general health.[34] This provides another possible reason for the increased SA among employees in lower prestige occupations.

We found increased IRR for SA days across all the intersectional groups compared with men in high prestige occupations, which was expected. Contrary to expectations, the IRR for SA days was higher for women in high prestige occupations than for women and men in lower prestige occupations. This finding supports the notion that some people occupy intersectional locations that include both privileges and marginalization.[35] The finding highlights the importance of the intersectional approach and the limitations associated with taking a unitary approach in evaluating health inequality.[36] The increased absence rate among women in high prestige occupations could reflect stressful conditions emanating from the combination of household chores and high workload. Berntsson *et al.*[37] found that women in high positions in Sweden are still mainly responsible for household tasks and childcare duties, and they reported higher levels of stressful conditions than men in similar positions.

In this study, among men, we found substantial occupational prestige differences in long-term SA, whereas among women, there was no such differences. In the intersectional analysis, men in lower prestige occupations also had higher odds for long-term SA than women regardless of prestige levels. Higher SA incidence in women than in men has been well supported in previous studies[20, 21] but longer absence spells among women has been a controversial topic in the literature, including in studies with similar cut-off points. For instance, Lidwall and Marklund[38] reported a higher risk for more than 60 absence days among women compared with men, whereas Laaksonen *et al.*[39] did not find increased



For peer review only - http://bmjopen.bmj.com/site/about/guidelines.xhtml

female absence using a cut-off point of 60 days. In Sweden[40] and abroad,[41] longer absence duration has been reported among men compared with women, which aligns with our finding of increased risk among men in lower prestige occupations. A possible explanation for this finding may relate to men's value system. Men attribute significant importance to values, such as power, status, and achievement.[42] Because they are sensitive to being relegated to a subordinate position, men in lower status occupations may develop negative perceptions of themselves, and to compensate, may engage in unhealthy behaviours that may jeopardize their psychological health.[43] Peterson[44] found that how men perceived their level of control, authority, and status had a significant impact on their psychological wellbeing, and psychiatric illness has been reported previously as a determinant of long-term SA among men in Sweden.[40] Research has also shown that men are more likely to dismiss their health needs and delay seeking health care compared with women;[45] such behaviours may worsen health situations and lead to long-term SA. The tendency of women to be more proactive in consulting professional help, may, on the one hand, explain their increased absenteeism rates, and, on the other hand, provide an alternative explanation for their decreased risk for long-term SA as observed in the current study.

The strengths of the current study include the longitudinal design, the use of register-based SA data, and the relatively long follow-up (observation period covering 2005–2013), which spanned periods of varying economic situations. The use of GEE made it possible to account for within-individual correlation in the SA variable, which is not attainable using ordinary regression methods. The application of the intersectional approach can be considered as an extension of previous studies on gender differences in SA, because it provided deeper insights on how gender and occupational prestige are mutually constituted to shape SA inequality in the working population.[23] This study was based on data of a large, reliable nationally representative sample (>70% LFS response rate) of employed women and



For peer review only - http://bmjopen.bmj.com/site/about/guidelines.xhtml

men, making it possible for the findings to be generalized to the Swedish working population. One limitation of the study relates to our measure of occupational prestige. The measure was based on occupational titles provided in the inclusion year, which may not necessarily be the longest held occupations for some of the participants. Due to reliance on occupational data provided in the inclusion year, we could not assess whether a participant changed occupations during follow-up and whether such changes might have any influence on the associations. Previous research has shown increased levels of physical workload among employees in lower social positions (measured as a combination of educational level and occupational groups) that consequently was associated with increased risk of SA.[46] For lack of data, we could not assess the influence of physical workload on the association between occupational prestige and SA. We could not also assess the influence of work-related psychosocial factors (work demand, control, and support), which have been associated with occupational prestige[30] and SA.[47] However, we did include several employment-related factors (contract type, employment sector, and employment type) as proxy measures for these psychosocial work characteristics, and our findings seem not to be influenced by these factors. Our measure of SA did not include absence spells lasting 14 days or less. We also did not examine cause-specific SA. It is possible that occupational prestige will exert varying effects on these forms of SA.

# CONCLUSIONS

This research demonstrated an association between lower occupational prestige and increased SA in women and men. Compared with men in high prestige occupations, women in all prestige levels and men in the lower prestige occupations had higher probability of SA days, with the highest IRR found among women in high prestige occupations. Men in low and medium prestige occupations had higher odds for long-term SA than the rest of the groups.



For peer review only - http://bmjopen.bmj.com/site/about/guidelines.xhtml

For peer review only

The findings highlight the importance of expanding discourses on SA inequality to include aspects of occupation that are rarely discussed, such as occupational prestige. Interventions that would help employees in low prestige occupations to valorise their occupations may be helpful in promoting health and wellbeing and reducing SA, especially among men.

**Acknowledgement:** We thank Ylva Ulfsdotter Eriksson for her constructive comments on this study.

**Funding:** This work was supported by [Forte] grant number [2016-07204].

**Competing interest**: None declared

**Author contributions:** CAN designed the study and was chiefly responsible for analysing the data and writing the manuscript drafts. GH participated in developing the research design, interpreting the results and writing the manuscript. TB delivered the data and contributed to the interpretation of the results and writing of the manuscript. All authors read and approved the final manuscript.

**Data availability statement**: The authors do not have the permission to share the data that support the findings of this study due to data protection regulations. Deidentified participant data are available from Statistics Sweden (scb@scb.se) on reasonable request.

# REFERENCES

1. Hultin H, Lindholm C, Möller J. Is there an association between long-term sick leave and disability pension and unemployment beyond the effect of health status?- a cohort study. *PloS One* 2012;7(4):e35614. Doi: 10.1371/journal.pone.0035614.
2. Salonen L, Blomgren J, Laaksonen M, et al. Sickness absence as a predictor of disability retirement in different occupational classes: a register-based study of a



For peer review only - http://bmjopen.bmj.com/site/about/guidelines.xhtml

working-age cohort in Finland in 2007-2014. *BMJ Open* 2018;8(5):e020491 doi:10.1136/bmjopen-2017-020491

3. Kivimäki M, Head J, Ferrie JE, et al. Sickness absence as a global measure of health: evidence from mortality in the Whitehall II prospective cohort study. *BMJ* 2003;327(7411):364. Doi:10.1136/bmj.327.7411.364
4. Connelly R, Gayle V, Lambert PS. A review of occupation-based social classifications for social survey research. *Methodological Innovations* 2016;9:1-14
5. Hansen H-T, Ingebrigtsen T. Social class and sickness absence in Norway. *Acta Sociologica 2008*;51(4)309-327
6. Piha K, Laaksonen M, Martikainen P, et al. Socio-economic and occupational determinants of work injury absence. *European Journal of Public Health* 2013;23(4):693-698
7. Kristensen TR, Jensen SM, Kreiner S, et al. Socioeconomic status and duration and pattern of sickness absence. A 1-year follow-up study of 2331 hospital employees. *BMC Public Health* 2010;10:643 http://www.biomecentral.com/1471-2458/10/643
8. Lambert PS, Bihagen E. Using occupation-based social classifications. *Work Employ Soc* 2014;28(3):481–94.
9. Treiman D. Occupational prestige in comparative perspective. New York: Wiley; 1977
10. Galobardes B, Shaw M, Lawlor DA, et al. Indicators of socioeconomic position (part 2). *J Epidemiol Community Health* 2006;60:95-101
11. Van Kleef GA, Cheng JT. Power, status, and hierarchy: current trends and future challenges. *Current Opinion in Psychology* 2020, 33: v-xiv. https://doi.org/10.1016/j.copsyc.2020.03.011



For peer review only - http://bmjopen.bmj.com/site/about/guidelines.xhtml

12. Ulfsdotter Eriksson Y, Linde M. ""Being" or "Doing" a profession: work as a matter of social identity". *The international Journal of Interdisciplinary Cultural Studies* 2014;8(1):33-43

13. Duemmler K, Caprani I. Identity strategies in light of a low prestige occupation: the case of retail apprentices. *J Educ Work* 2017;30(4):339–52.

14. Duemmler K, Caprani I, Felder A. The challenge of occupational prestige for occupational identities: comparing bricklaying and automation technology apprentices in Switzerland. *Vocations and Learning* 2020;13(3):369-388. Doi: 10.1007/s12186-020-09243-3

15. Marmot MG. Status syndrome a challenge to Medicine. *JAMA* 2006;295(11):1304–7.

16. Fournier MA. Dimensions of human hierarchy as determinants of health and happiness. *Current Opinion in Psychology* 2020;33:110-114

17. Fujishiro K, Xu J, Gong F. What does "occupation" represent as an indicator of socioeconomic status? Exploring occupational prestige and health. *Soc Sci Med* 2010;71:2100–7.

18. Halleröd B, Gustafsson J-E. A longitudinal analysis of the relationship between changes in socio-economic status and changes in health. *Soc Sci Med* 2011;72:116–23.

19. Christ SL, Fleming LE, Lee DJ, et al. The effects of a psychosocial dimension of socioeconomic position on survival: occupational prestige and mortality among US working adults. *Sociology of Health & Illness* 2012;34(7):1103–17.

20. Casini A, Godin I, Clays E, et al. Gender difference in sickness absence from work: a multiple mediation analysis of psychosocial factors. *European Journal of Public Health* 2013;23(4):635-642



For peer review only - http://bmjopen.bmj.com/site/about/guidelines.xhtml

21. Østby KA, Mykletun A, Nilsen W. Explaining the gender gap in sickness absence. *Occupational Medicine* 2018;68:320-326.

22. Hankivsky O, Reid C, Cormier R, et al. Exploring the promises of intersectionality for advancing women's health research. *Int J Equity Health* 2010;9:5. doi:10.1186/1475-9276-9-5

23. Green MA, Evans CR, Subramanian SV. Can intersectionality theory enrich population health research? *Soc Sci Med* 2017;178:214–16. doi:10.1016/j.socscimed.2017.02.029

24. Skans LH. Labor market development according to different statistical sources. *Economic Commentaries* 2019;6:1–12.

25. Ludvigsson JF, Svedberg P, Olén O, et al. The longitudinal integrated database for health insurance and labor market studies (LISA) and its use in medical research. *Eur J Epidemiol* 2019;34:423–37. doi:10.1007/s10654-019-00511-8.

26. Treiman DJ. A Standard Occupational prestige scale for use with historical data. *The Journal of Interdisciplinary History* 1976;7(2):283-304.

27. Svensson LG, Ulfsdotter EY. Yrkesstatus. En sociologisk studie av hur yrken uppfattas och värderas [Occupational Status. A Sociological Study on the perceptions and valuations of occupations]. Research report no. 140 from the Department of Sociology, Gothenburg University.

28. Liang K-Y, Zeger SL. Longitudinal data analysis using generalized linear models. *Biometrika* 1986;73:13–22. doi:10.1093/biomet/73.1.13

29. Ballinger GA. Using generalized estimating equations for longitudinal data analysis. *Organ Res Methods* 2004;7(2):127–50. doi:10.1177/1094428104263672



For peer review only - http://bmjopen.bmj.com/site/about/guidelines.xhtml

30. Hoven H, Wahrendorf M, Siegrist J. Occupational position, work stress and depressive symptoms: a pathway analysis of longitudinal SHARE data. *J Epidemiol Community Health* 2015;69:447–52. doi:10.1136/jech-2014-205206

31. Mann M, Hosman CMH, Schealma HP, et al. Self-esteem in a broad-spectrum approach for mental health promotion. *Health Educ Res* 2004;19(4):357–72. doi:10.1093/her/cyg041

32. Yanos PT. The impact of illness identity on recovery from severe mental illness. *Am J Psychiatr Rehabil* 2010;13(2):73–93. doi:10.1080/15487761003756860

33. Matthews KA, Räikkönen K, Everson SA, et al. Do the daily experiences of healthy men and women vary according to occupational prestige and work strain? *Psychosom Med* 2000;62:346–53. doi:10.1097/00006842-200005000-00008

34. De Raeve L, Jansen NWH, van den Brandt PA, et al. Interpersonal conflicts at work as a predictor of self-reported health outcomes and occupational mobility. *Occup Environ Med* 2007;66:16–22. doi:10.1136/oem.2007.037655

35. Haukenes I, Löve J, Hensing G, et al. Inequality in disability: an intersectional analysis of the co-constitution of gender, education and age. The Hordaland Health Study. *Crit Public Health* 2018;29(3):302–13. doi:10.1080/09581596.2018.1469730

36. Bauer GR. Incorporating intersectionality theory into population health research methodology: challenges and the potential to advance health equity. *Soc Sci Med* 2014;110:10–17. doi:10.1016/j.socscimed.2014.03.022

37. Berntsson L, Lundberg U, Krantz G. Gender differences in work-home interplay and symptom perception among Swedish white-collar employees. *J Epidemiol Community Health* 2006;60:1070–6. doi:10.1136/jech.2005.042192



For peer review only - http://bmjopen.bmj.com/site/about/guidelines.xhtml

38. Lidwall U, Marklund S. What is health work for women and men? A case-control study of gender-and sector-specific effects of psycho-social working conditions on long-term sickness absence. *Work* 2006;27(2):153–63.

39. Laaksonen M, Martikainen P, Rahkonen O, et al. Explanations for gender differences in sickness absence: evidence from middle-aged municipal employees from Finland. *Occup Environ Med* 2008;65:325–30. doi:10.1136/oem.2007.033910

40. Hensing G, Alexanderson K, Allebeck P, et al. Sick leave due to psychiatric disorder: higher incidence among women and longer duration for men. *Br J Psychiatry* 1996;169:740–6. doi:10.1192/bjp.169.6.740

41. Sandanger I, Nygård JF, Brage S, et al. Relation between health problems and sickness absence: Gender and age differences. A comparison of low-back pain, psychiatric disorders and injuries. *Scand J Public Health* 2000;28:244–52. doi:10.1177/140349480002800406011

42. Schwartz SH, Lifschitz TR. Sex differences in value priorities: cross-cultural and multimethod studies. *Journal of Personality and Social Psychology* 2006;89(6):1010-28. Doi: 10.1037/0022-3514.89.6.1010

43. Courtenay WH. Constructions of masculinity and their influence on men's well-being: a theory of gender and health. *Soc Sci Med* 2000;50(10):1385–401. doi:10.1016/S0277-9536(99)00390-1

44. Peterson M. What men and women value at work: implications for workplace health. *Gender Med* 2004;1(2):106–24. doi:10.1016/S1550-8579(04)80016-0

45. Thompson AE, Anisimowicz Y, Miedema B, et al. The influence of gender and other patient characteristics on health care-seeking behavior: a QUALICOPC study. *BMC Fam Pract* 2016;17:38. doi:10.1186/s12875-016-0440-0



For peer review only - http://bmjopen.bmj.com/site/about/guidelines.xhtml

46. Corbett K, Gran JM, Kristensen P, et al. Adult social position and sick leave: the mediating effect of physical workload. *Scand J Work Environ Health* 2015;41(6):542-553

47. Slany C, Schütte S, Chastang J-F, et al. Psychosocial work factors and long sickness absence in Europe. *Int J Occup Environ Health* 2014;20(1):16–25. doi:10.1179/2049396713Y.0000000048

For peer review only

Figure 1. Flowchart illustrating selection of study participants. LFS, labour force survey

For peer review only



For peer review only - http://bmjopen.bmj.com/site/about/guidelines.xhtml

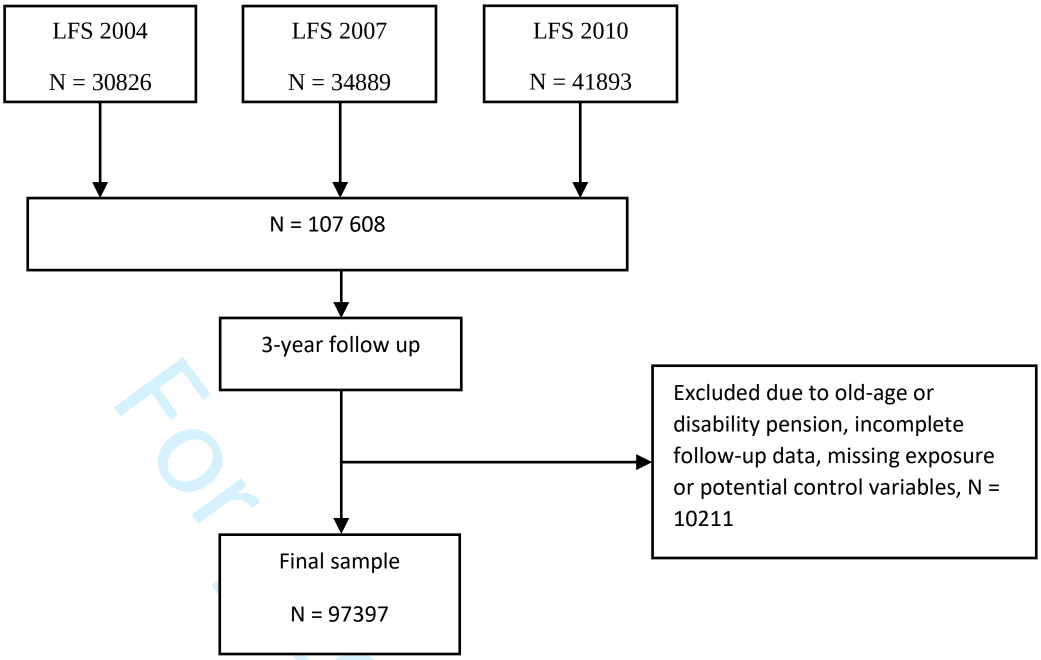


Figure 1. Flowchart illustrating selection of study participants. LFS, labour force survey

For peer review only

Online supplementary table A1. Gender-stratified association between occupational prestige and number of sickness absence days at follow-up. Incidence rate ratios (IRR) and 95% confidence intervals (95%CI) obtained from generalized estimating equation (GEE) with negative binomial regression

| | | Age-adjusted<br>IRR (95%CI) | Model I<br>IRR (95%CI) | Model II<br>IRR (95%CI) | Model III<br>IRR (95%CI) | Model IV<br>IRR (95%CI) | Model V<br>IRR (95%CI) |
|---|---|---|---|---|---|---|---|
| Women | N = 48029 weighted % | | | | | | |
| Occupational prestige | | | | | | | |
| Low | 34.8 | 2.05 (2.02-2.08) | 2.06 (2.03-2.09) | 1.85 (1.82-1.88) | 1.48 (1.45-1.51) | 1.24 (1.19-1.28) | 1.22 (1.18-1.27) |
| Medium | 32.1 | 1.21 (1.19-1.23) | 1.19 (1.18-1.21) | 1.12 (1.11-1.14) | 1.01 (0.99-1.03) | 0.99 (0.97-1.01) | 1.05 (1.02-1.07) |
| High | 33.1 | 1.00 | 1.00 | 1.00 | 1.00 | 1.00 | 1.00 |
| Men | N = 49368 | | | | | | |

For peer review only

| | weighted % | | | | | | |
|---|---|---|---|---|---|---|---|
| Occupational prestige | | | | | | | |
| Low | 30.4 | 3.61 (3.52-3.70) | 3.56 (3.47-3.64) | 3.08 (3.01-3.16) | 1.55 (1.50-1.60) | 0.97 (0.94-1.01) | 1.00 (0.96-1.05) |
| Medium | 38.9 | 2.15 (2.10-2.21) | 2.15 (2.10-2.20) | 2.05 (2.00-2.11) | 1.21 (1.17-1.25) | 0.88 (0.85-0.91) | 0.92 (0.89-0.95) |
| High | 30.7 | 1.00 | 1.00 | 1.00 | 1.00 | 1.00 | 1.00 |

Model I, additionally adjusting for survey year and marital status; Model II, additionally adjusting for previous sickness; Model III, additionally adjusting for education and income; Model IV, additionally adjusting for occupational class; Model V, additionally adjusting for employment type, contract type, and employment sector

For peer review only

Online supplementary table 2. Association of occupational prestige with long-term sickness absence among those with at least one spell of sickness absence. Odds ratios (OR) and 95% confidence intervals (95%CI) obtained from generalized estimating equation (GEE) with logistic regression

| | Total N = 21836 | Age-adjusted | Model I | Model II | Model III | Model IV | Model V |
|---|---|---|---|---|---|---|---|
| | weighted % | OR (95%CI) | OR (95%CI) | OR (95%CI) | OR (95%CI) | OR (95% CI) | OR (95% CI) |
| Occupational prestige | | | | | | | |
| Low | 43.4 | 1.51 (1.48-1.54) | 1.51 (1.48-1.54) | 1.38 (1.36-1.41) | 1.03 (1.00-1.05) | 1.08 (1.04-1.11) | 1.07 (1.04-1.11) |
| Medium | 33.4 | 1.11 (1.08-1.13) | 1.12 (1.09-1.14) | 1.07 (1.04-1.09) | 0.91 (0.89-0.93) | 0.98 (0.96-1.01) | 1.00 (0.98-1.03) |
| High | 23.2 | 1.00 | 1.00 | 1.00 | 1.00 | 1.00 | 1.00 |

Model I, additionally adjusting for survey year, gender, and marital status; Model II, additionally adjusting for previous sickness; Model III, additionally adjusting for education and income; Model IV, additionally adjusting for occupational class; Model V, additionally adjusting for employment type, contract type, and employment sector

For peer review only

Online supplementary table A3. Intersections of gender and occupational prestige and the association with long-term sickness absence among those with at least one spell of sickness absence. Odds ratios (OR) and 95% confidence intervals (95%CI) obtained from generalized estimating equation (GEE) with logistic regression

| | Total N = 21836 | Age-adjusted | Model I | Model II | Model III | Model IV | Model V |
|---|---|---|---|---|---|---|---|
| | weighted % | OR (95%CI) | OR (95%CI) | OR (95% CI) | OR (95% CI) | OR (95%CI) | OR (95%CI) |
| Women/low prestige occup. | 26.7 | 2.09 (2.01-2.16) | 2.08 (2.00-2.15) | 1.70 (1.64-1.77) | 1.05 (1.00-1.09) | 1.11 (1.06-1.16) | 1.05 (1.00-1.11) |
| Women/medium prestige occup. | 18.1 | 1.53 (1.47-1.59) | 1.51 (1.46-1.57) | 1.30 (1.25-1.35) | 0.93 (0.89-0.96) | 1.02 (0.98-1.06) | 1.02 (0.98-1.06) |
| Women/high prestige occup. | 16.3 | 1.49 (1.43-1.55) | 1.49 (1.44-1.55) | 1.32 (1.27-1.37) | 1.08 (1.04-1.12) | 1.09 (1.05-1.14) | 1.06 (1.02-1.11) |
| Men/low prestige occup. | 16.7 | 1.92 (1.86-2.00) | 1.87 (1.80-1.94) | 1.67 (1.61-1.73) | 1.22 (1.17-1.27) | 1.30 (1.24-1.37) | 1.31 (1.25-1.38) |
| Men/medium prestige occup. | 15.3 | 1.43 (1.37-1.87) | 1.41 (1.35-1.46) | 1.30 (1.25-1.35) | 1.08 (1.03-1.12) | 1.16 (1.11-1.21) | 1.19 (1.13-1.24) |
| Men/high prestige occup. | 6.8 | 1.00 | 1.00 | 1.00 | 1.00 | 1.00 | 1.00 |

Model I, additionally adjusting for survey year and marital status; Model II, additionally adjusting for previous sickness; Model III, additionally adjusting for education and income; Model IV, additionally adjusting for occupational class; Model V, additionally adjusting for employment type, contract type, and employment sector

For peer review only

**STROBE 2007 (v4) Statement—Checklist of items that should be included in reports of *cohort studies***

| Section/Topic | Item # | Recommendation | Reported on page # |
|---|---|---|---|
| **Title and abstract** | 1 | (*a*) Indicate the study's design with a commonly used term in the title or the abstract | 2 |
| | | (*b*) Provide in the abstract an informative and balanced summary of what was done and what was found | 2-3 |
| **Introduction** | | | |
| Background/rationale | 2 | Explain the scientific background and rationale for the investigation being reported | 5-7 |
| Objectives | 3 | State specific objectives, including any prespecified hypotheses | 7 |
| **Methods** | | | |
| Study design | 4 | Present key elements of study design early in the paper | 8 |
| Setting | 5 | Describe the setting, locations, and relevant dates, including periods of recruitment, exposure, follow-up, and data collection | 7-8 |
| Participants | 6 | (*a*) Give the eligibility criteria, and the sources and methods of selection of participants. Describe methods of follow-up | 8 |
| | | (*b*) For matched studies, give matching criteria and number of exposed and unexposed | |
| Variables | 7 | Clearly define all outcomes, exposures, predictors, potential confounders, and effect modifiers. Give diagnostic criteria, if applicable | 9-11 |
| Data sources/ measurement | 8* | For each variable of interest, give sources of data and details of methods of assessment (measurement). Describe comparability of assessment methods if there is more than one group | 9-11 |
| Bias | 9 | Describe any efforts to address potential sources of bias | 14 |
| Study size | 10 | Explain how the study size was arrived at | 8 |
| Quantitative variables | 11 | Explain how quantitative variables were handled in the analyses. If applicable, describe which groupings were chosen and why | 9-11 |
| Statistical methods | 12 | (*a*) Describe all statistical methods, including those used to control for confounding | 13-14 |
| | | (*b*) Describe any methods used to examine subgroups and interactions | |
| | | (*c*) Explain how missing data were addressed | 8 |
| | | (*d*) If applicable, explain how loss to follow-up was addressed | 8 |
| | | (*e*) Describe any sensitivity analyses | 14 |
| **Results** | | | |

For peer review only

| | | | |
|---|---|---|---|
| Participants | 13* | (a) Report numbers of individuals at each stage of study—eg numbers potentially eligible, examined for eligibility, confirmed eligible, included in the study, completing follow-up, and analysed | |
| | | (b) Give reasons for non-participation at each stage | |
| | | (c) Consider use of a flow diagram | |
| Descriptive data | 14* | (a) Give characteristics of study participants (eg demographic, clinical, social) and information on exposures and potential confounders | 16-19 |
| | | (b) Indicate number of participants with missing data for each variable of interest | |
| | | (c) Summarise follow-up time (eg, average and total amount) | 20 |
| Outcome data | 15* | Report numbers of outcome events or summary measures over time | 20 |
| Main results | 16 | (*a*) Give unadjusted estimates and, if applicable, confounder-adjusted estimates and their precision (eg, 95% confidence interval). Make clear which confounders were adjusted for and why they were included | 23-32 |
| | | (*b*) Report category boundaries when continuous variables were categorized | |
| | | (*c*) If relevant, consider translating estimates of relative risk into absolute risk for a meaningful time period | |
| Other analyses | 17 | Report other analyses done—eg analyses of subgroups and interactions, and sensitivity analyses | 28 |
| **Discussion** | | | |
| Key results | 18 | Summarise key results with reference to study objectives | 33 |
| **Limitations** | | | |
| Interpretation | 20 | Give a cautious overall interpretation of results considering objectives, limitations, multiplicity of analyses, results from similar studies, and other relevant evidence | 33-36 |
| Generalisability | 21 | Discuss the generalisability (external validity) of the study results | 36 |
| **Other information** | | | |
| Funding | 22 | Give the source of funding and the role of the funders for the present study and, if applicable, for the original study on which the present article is based | 37 |

*Give information separately for cases and controls in case-control studies and, if applicable, for exposed and unexposed groups in cohort and cross-sectional studies.

**Note:** An Explanation and Elaboration article discusses each checklist item and gives methodological background and published examples of transparent reporting. The STROBE checklist is best used in conjunction with this article (freely available on the Web sites of PLoS Medicine at http://www.plosmedicine.org/, Annals of Internal Medicine at http://www.annals.org/, and Epidemiology at http://www.epidem.com/). Information on the STROBE Initiative is available at www.strobe-statement.org.

For peer review only
